# Supplementary material for: Increase of Synergistic Secondary Antiviral Mutations in the Evolution of A(H1N1)pdm09 Influenza Virus Neuraminidases
Source: Viruses. 2024 Jul 11;16(7):1109. doi: 10.3390/v16071109 (PMC11281601; doi:10.3390/v16071109)
Supplement: Supplementary file 1 [file viruses-16-01109-s001.zip › viruses-3070662-supplementary.pdf]

Table S1. Accession number (Gisaid) of influenza A(H1N1)pdm09 viruses isolated in Germany between 2009 and 2024

EPI_ISL_73769	A/Sachsen-Anhalt/138/2009	EPI_ISL_19140819	A/Baden-Wuerttemberg/4/2010
EPI_ISL_73768	A/Sachsen-Anhalt/137/2009	EPI_ISL_19140820	A/Baden-Wuerttemberg/8/2010
EPI_ISL_73766	A/Sachsen-Anhalt/134/2009	EPI_ISL_19140821	A/Baden-Wuerttemberg/12/2009
EPI_ISL_73764	A/Sachsen-Anhalt/121/2009	EPI_ISL_19140822	A/Baden-Wuerttemberg/10/2009
EPI_ISL_73763	A/Sachsen-Anhalt/117/2009	EPI_ISL_19140823	A/Baden-Wuerttemberg/10/2010
EPI_ISL_73760	A/Sachsen/180/2009	EPI_ISL_19140824	A/Baden-Wuerttemberg/11/2009
EPI_ISL_73759	A/Sachsen/156/2009	EPI_ISL_19140825	A/Bayern/73/2009
EPI_ISL_73750	A/Niedersachsen/92/2009	EPI_ISL_19140826	A/Bayern/74/2009
EPI_ISL_73741	A/Niedersachsen/336/2009	EPI_ISL_19140827	A/Bayern/63/2009
EPI_ISL_73739	A/Niedersachsen/216/2009	EPI_ISL_19140828	A/Bayern/69/2009
EPI_ISL_73738	A/Niedersachsen/188/2009	EPI_ISL_19140829	A/Bayern/77/2009
EPI_ISL_73737	A/Niedersachsen/134/2009	EPI_ISL_19140830	A/Bayern/78/2009
EPI_ISL_73736	A/Niedersachsen/109/2009	EPI_ISL_19140831	A/Bayern/84/2009
EPI_ISL_73735	A/Niedersachsen/104/2009	EPI_ISL_19140832	A/Bayern/86/2009
EPI_ISL_73689	A/Bayern/79/2009	EPI_ISL_19140833	A/Bayern/96/2009
EPI_ISL_73710	A/Berlin/97/2009	EPI_ISL_19140834	A/Bayern/107/2009
EPI_ISL_73698	A/Brandenburg/43/2009	EPI_ISL_19140835	A/Bayern/150/2009
EPI_ISL_73742	A/Niedersachsen/343/2009	EPI_ISL_19140836	A/Bayern/154/2009
EPI_ISL_73743	A/Niedersachsen/352/2009	EPI_ISL_19140837	A/Bayern/125/2009
EPI_ISL_19140806	A/Schleswig-Holstein/16/2009	EPI_ISL_19140838	A/Bayern/128/2009
EPI_ISL_19140807	A/Bayern/75/2009	EPI_ISL_19140839	A/Bayern/117/2009
EPI_ISL_19140808	A/Hessen/47/2009	EPI_ISL_19140840	A/Bayern/9/2010
EPI_ISL_19140809	A/Nordrhein-Westfalen/74/2009	EPI_ISL_19140814	A/Baden-Wuerttemberg/487/2009
EPI_ISL_19140810	A/Hessen/48/2009	EPI_ISL_19140815	A/Baden-Wuerttemberg/487/2009
EPI_ISL_19140811	A/Baden-Wuerttemberg/459/2009	EPI_ISL_19140816	A/Baden-Wuerttemberg/490/2009
EPI_ISL_19140812	A/Baden-Wuerttemberg/462/2009	EPI_ISL_19140817	A/Baden-Wuerttemberg/502/2009
EPI_ISL_19140813	A/Baden-Wuerttemberg/464/2009	EPI_ISL_19140818	A/Baden-Wuerttemberg/511/2009
EPI_ISL_19140841	A/Berlin/74/2009	EPI_ISL_19140873	A/Hessen/10/2009
EPI_ISL_19140842	A/Berlin/72/2009	EPI_ISL_19140874	A/Mecklenburg-Vorpommern/1/2009
EPI_ISL_19140843	A/Berlin/80/2009	EPI_ISL_19140875	A/Mecklenburg-Vorpommern/6/2009
EPI_ISL_19140844	A/Berlin/78/2009	EPI_ISL_19140876	A/Mecklenburg-Vorpommern/10/09
EPI_ISL_19140845	A/Berlin/79/2009	EPI_ISL_19140877	A/Mecklenburg-Vorpommern/11/2009

Table S1. Accession number (Gisaid) of influenza A(H1N1)pdm09 viruses isolated in Germany between 2009 and 2024

EPI_ISL_19140846	A/Berlin/75/2009	EPI_ISL_19140878	A/Mecklenburg-Vorpommern/10/2009
EPI_ISL_19140847	A/Berlin/87/2009	EPI_ISL_19140879	A/Niedersachsen/339/2009
EPI_ISL_19140848	A/Berlin/88/2009	EPI_ISL_19140880	A/Niedersachsen/84/2009
EPI_ISL_19140849	A/Berlin/90/2009	EPI_ISL_19140881	A/Niedersachsen/87/2009
EPI_ISL_19140850	A/Berlin/83/2009	EPI_ISL_19140882	A/Niedersachsen/88/2009
EPI_ISL_19140851	A/Berlin/121/2009	EPI_ISL_19140883	A/Niedersachsen/415/2009
EPI_ISL_19140852	A/Berlin/130/2009	EPI_ISL_19140884	A/Niedersachsen/10/2010
EPI_ISL_19140853	A/Berlin/147/2009	EPI_ISL_19140885	A/Nordrhein-Westfalen/100/2009
EPI_ISL_19140854	A/Berlin/164/2009	EPI_ISL_19140886	A/Nordrhein-Westfalen/87/2009
EPI_ISL_19140855	A/Berlin/185/2009	EPI_ISL_19140887	A/Nordrhein-Westfalen/94/2009
EPI_ISL_19140856	A/Berlin/187/2009	EPI_ISL_19140888	A/Nordrhein-Westfalen/99/2009
EPI_ISL_19140857	A/Berlin/210/2009	EPI_ISL_19140889	A/Nordrhein-Westfalen/103/2009
EPI_ISL_19140858	A/Berlin/10/2009	EPI_ISL_19140890	A/Nordrhein-Westfalen/106/2009
EPI_ISL_19140859	A/Berlin/10/2010	EPI_ISL_19140891	A/Nordrhein-Westfalen/113/2009
EPI_ISL_19140860	A/Berlin/143/2009	EPI_ISL_19140892	A/Nordrhein-Westfalen/137/2009
EPI_ISL_19140861	A/Brandenburg/38/2009	EPI_ISL_19140893	A/Nordrhein-Westfalen/152/2009
EPI_ISL_19140862	A/Brandenburg/28/2009	EPI_ISL_19140894	A/Nordrhein-Westfalen/184/2009
EPI_ISL_19140863	A/Brandenburg/37/2009	EPI_ISL_19140895	A/Nordrhein-Westfalen/163/2009
EPI_ISL_19140864	A/Brandenburg/42/2009	EPI_ISL_19140896	A/Nordrhein-Westfalen/201/2009
EPI_ISL_19140865	A/Brandenburg/24/2009	EPI_ISL_19140897	A/Rheinland-Pfalz/43/2009
EPI_ISL_19140866	A/Brandenburg/34/2009	EPI_ISL_19140898	A/Rheinland-Pfalz/41/2009
EPI_ISL_19140867	A/Brandenburg/10/2009	EPI_ISL_19140899	A/Rheinland-Pfalz/44/2009
EPI_ISL_19140868	A/Bremen/18/2009	EPI_ISL_19140900	A/Rheinland-Pfalz/81/2009
EPI_ISL_19140869	A/Bremen/20/2009	EPI_ISL_19140901	A/Rheinland-Pfalz/86/2009
EPI_ISL_19140870	A/Hamburg/6/2009	EPI_ISL_19140902	A/Rheinland-Pfalz/10/2009
EPI_ISL_19140871	A/Hamburg/14/2009	EPI_ISL_19140903	A/Rheinland-Pfalz/11/2009
EPI_ISL_19140872	A/Hessen/61/2009	EPI_ISL_19140904	A/Saarland/3/2009
EPI_ISL_19140905	A/Saarland/16/2009	EPI_ISL_19140936	A/Nordrhein-Westfalen/173/2009
EPI_ISL_19140906	A/Sachsen/96/2009	EPI_ISL_19140937	A/Nordrhein-Westfalen/174/2009
EPI_ISL_19140907	A/Sachsen/93/2009	EPI_ISL_19140938	A/Nordrhein-Westfalen/175/2009
EPI_ISL_19140908	A/Sachsen/92/2009	EPI_ISL_19140939	A/Niedersachsen/850/2009
EPI_ISL_19140909	A/Sachsen/10/2009	EPI_ISL_19140940	A/Niedersachsen/866/2009

Table S1. Accession number (Gisaid) of influenza A(H1N1)pdm09 viruses isolated in Germany between 2009 and 2024

EPI_ISL_19140910	A/Sachsen/97/2009	EPI_ISL_19140941	A/Sachsen/183/2009
EPI_ISL_19140911	A/Sachsen/129/2009	EPI_ISL_19140942	A/Sachsen-Anhalt/178/2009
EPI_ISL_19140912	A/Sachsen/154/2009	EPI_ISL_19141140	*A/Berlin/12/2010*
EPI_ISL_19140913	A/Sachsen/194/2009	EPI_ISL_19141141	*A/Berlin/14/2010*
EPI_ISL_19140969	A/Sachsen/194/2009 Res	EPI_ISL_19141142	*A/Berlin/15/2010*
EPI_ISL_19140914	A/Sachsen/4/2010	EPI_ISL_19141143	*A/Berlin/17/2010*
EPI_ISL_19140915	A/Sachsen-Anhalt/9/2010	EPI_ISL_19141144	*A/Berlin/18/2010*
EPI_ISL_19140916	A/Schleswig-Holstein/13/2009	EPI_ISL_19141189	A/Baden-Wuerttemberg/13/2010
EPI_ISL_19140917	A/Thueringen/173/2009	EPI_ISL_19141190	A/Baden-Wuerttemberg/14/2010
EPI_ISL_19140918	A/Thueringen/176/2009	EPI_ISL_19141191	A/Nordrhein-Westfalen/11/2010
EPI_ISL_19140919	A/Thueringen/189/2009	EPI_ISL_19141145	A/Bayern/4/2011
EPI_ISL_19140920	A/Thueringen/201/2009	EPI_ISL_19141146	A/Bayern/7/2011
EPI_ISL_19140921	A/Thueringen/227/2009	EPI_ISL_19141147	A/Berlin/14/2011
EPI_ISL_19140922	A/Sachsen-Anhalt/127/2009	EPI_ISL_19141148	A/Bayern/39/2011
EPI_ISL_19140923	A/Sachsen-Anhalt/135/2009	EPI_ISL_19141149	A/Brandenburg/1/2011
EPI_ISL_19140924	A/Niedersachsen/330/2009	EPI_ISL_19141150	A/Berlin/6/2011
EPI_ISL_19140925	A/Sachsen-Anhalt/144/2009	EPI_ISL_19141151	A/Berlin/8/2011
EPI_ISL_19140926	A/Niedersachsen/115/2009	EPI_ISL_19141152	A/Berlin/23/2011
EPI_ISL_19140927	A/Sachsen-Anhalt/125/2009	EPI_ISL_19141153	A/Berlin/38/2011
EPI_ISL_19140928	A/Sachsen-Anhalt/119/2009	EPI_ISL_19141154	A/Bremen/3/2011
EPI_ISL_19140929	A/Sachsen-Anhalt/122/2009	EPI_ISL_19141155	A/Hamburg/3/2011
EPI_ISL_19140930	A/Niedersachsen/337/2009	EPI_ISL_19141156	A/Hessen/3/2011
EPI_ISL_19140931	A/Niedersachsen/338/2009	EPI_ISL_19141192	A/Nordrhein-Westfalen/5/2011
EPI_ISL_19140932	A/Sachsen-Anhalt/124/2009	EPI_ISL_19141193	A/Nordrhein-Westfalen/6/2011
EPI_ISL_19140933	A/Niedersachsen/91/2009	EPI_ISL_19141194	A/Nordrhein-Westfalen/16/2011
EPI_ISL_19140934	A/Nordrhein-Westfalen/171/2009	EPI_ISL_19141195	A/Nordrhein-Westfalen/53/2011
EPI_ISL_19140935	A/Nordrhein-Westfalen/172/2009	EPI_ISL_19141157	A/Niedersachsen/7/2011
EPI_ISL_19141158	A/Niedersachsen/15/2011	EPI_ISL_19141200	A/Nordrhein-Westfalen/14/2010
EPI_ISL_19141159	A/Niedersachsen/16/2011	EPI_ISL_19141183	A/Niedersachsen/11/2011
EPI_ISL_19141160	A/Niedersachsen/80/2011	EPI_ISL_19141184	A/Niedersachsen/13/2010
EPI_ISL_19141161	A/Rheinland-Pfalz/5/2011	EPI_ISL_19141185	A/Niedersachsen/11/2010
EPI_ISL_19141162	A/Rheinland-Pfalz/7/2011	EPI_ISL_19141186	A/Niedersachsen/12/2010

Table S1. Accession number (Gisaid) of influenza A(H1N1)pdm09 viruses isolated in Germany between 2009 and 2024

EPI_ISL_19141163	A/Rheinland-Pfalz/42/2011	EPI_ISL_19141187	A/Sachsen/28/2011
EPI_ISL_19141164	A/Rheinland-Pfalz/55/2011	EPI_ISL_19141188	A/Sachsen/10/2011
EPI_ISL_19141165	A/Saarland/1/2011	EPI_ISL_19141201	A/Rheinland-Pfalz/17/2011
EPI_ISL_19141166	A/Sachsen/2/2011	EPI_ISL_19141214	A/Baden-Wuerttemberg/60/2012
EPI_ISL_19141167	A/Sachsen/8/2011	EPI_ISL_19141215	A/Bayern/28/2012
EPI_ISL_19141168	A/Schleswig-Holstein/3/2011	EPI_ISL_19141216	A/Niedersachsen/10/2012
EPI_ISL_19141169	A/Thueringen/2/2011	EPI_ISL_19141217	A/Berlin/136/2012
EPI_ISL_19141170	A/Bayern/10/2010	EPI_ISL_19141218	A/Baden-Wuerttemberg/172/2012
EPI_ISL_19141171	A/Bayern/11/2010	EPI_ISL_19141219	A/Bayern/86/2012
EPI_ISL_19141172	A/Berlin/13/2011	EPI_ISL_19141220	A/Baden-Wuerttemberg/28/2012
EPI_ISL_19141173	A/Berlin/10/2011	EPI_ISL_19141221	A/Baden-Wuerttemberg/109/2012
EPI_ISL_19141174	A/Berlin/11/2010	EPI_ISL_19141222	A/Baden-Wuerttemberg/149/2012
EPI_ISL_19141142	A/Berlin/15/2010	EPI_ISL_19141223	A/Niedersachsen/6/2012
EPI_ISL_19141140	A/Berlin/12/2010	EPI_ISL_19141224	A/Baden-Wuerttemberg/125/2012
EPI_ISL_19141175	A/Berlin/11/2011	EPI_ISL_19141438	A/Bayern/91/2012
EPI_ISL_19141176	A/Berlin/12/2011	EPI_ISL_19141439	A/Berlin/167/2012
EPI_ISL_19141177	A/Berlin/13/2010	EPI_ISL_19141440	A/Nordrhein-Westfalen/38/2012
EPI_ISL_19141141	A/Berlin/14/2010	EPI_ISL_19141441	A/Baden-Wuerttemberg/80/2013
EPI_ISL_19141178	A/Berlin/16/2010	EPI_ISL_19141442	A/Hessen/4/2013
EPI_ISL_19141179	A/Bremen/2/2010	EPI_ISL_19141443	A/Rheinland-Pfalz/2/2013
EPI_ISL_19141180	A/Bremen/3/2010	EPI_ISL_19141444	A/Sachsen/6/2013
EPI_ISL_19141196	A/Baden-Wuerttemberg/10/2011	EPI_ISL_19141445	A/Bayern/93/2012
EPI_ISL_19141197	A/Baden-Wuerttemberg/11/2011	EPI_ISL_19141446	A/Thueringen/42/2012
EPI_ISL_19141198	A/Baden-Wuerttemberg/12/2011	EPI_ISL_19151069	A/Brandenburg/1/2014
EPI_ISL_19141199	A/Baden-Wuerttemberg/11/2010	EPI_ISL_19151057	A/Berlin/6/2014
EPI_ISL_19141181	A/Hessen/11/2011	EPI_ISL_19151058	A/Berlin/30/2014
EPI_ISL_19141182	A/Hessen/10/2011	EPI_ISL_19151059	A/Schleswig-Holstein/2/2014
EPI_ISL_19151060	A/Berlin/54/2014	EPI_ISL_19151093	A/Niedersachsen/68/2015
EPI_ISL_19151061	A/Berlin/55/2014	EPI_ISL_19151094	A/Niedersachsen/72/2015
EPI_ISL_19151062	A/Berlin/40/2014	EPI_ISL_19151095	A/Rheinland-Pfalz/53/2015
EPI_ISL_19151063	A/Berlin/10/2014	EPI_ISL_19151096	A/Rheinland-Pfalz/54/2015
EPI_ISL_19151064	A/Bayern/12/2014	EPI_ISL_19151097	A/Rheinland-Pfalz/10/2015

Table S1. Accession number (Gisaid) of influenza A(H1N1)pdm09 viruses isolated in Germany between 2009 and 2024

EPI_ISL_19151065	A/Sachsen/10/14	EPI_ISL_19151098	A/Thueringen/43/2015
EPI_ISL_19151066	A/Sachsen/10/2014	EPI_ISL_202714	A/Bayern/146/2015
EPI_ISL_19151070	A/Baden-Wuerttemberg/10/2014	EPI_ISL_208323	A/Bayern/148/2015
EPI_ISL_19151067	A/Berlin/13/2014	EPI_ISL_203628	A/Bayern/149/2015
EPI_ISL_19151068	A/Brandenburg/10/2014	EPI_ISL_208333	A/Bayern/151/2015
EPI_ISL_19151071	A/Berlin/11/2014	EPI_ISL_202562	A/Berlin/166/2015
EPI_ISL_19151072	A/Bremen/8/2014	EPI_ISL_202715	A/Berlin/167/2015
EPI_ISL_19151073	A/Berlin/12/2014	EPI_ISL_203623	A/Berlin/170/2015
EPI_ISL_19151074	A/Berlin/81/2014	EPI_ISL_203624	A/Berlin/171/2015
EPI_ISL_19151075	A/Hamburg/7/2014	EPI_ISL_208355	A/Berlin/174/2015
EPI_ISL_19151076	A/Rheinland-Pfalz/12/2014	EPI_ISL_208327	A/Berlin/175/2015
EPI_ISL_19151077	A/Rheinland-Pfalz/14/2014	EPI_ISL_208322	A/Baden-Wuerttemberg/253/2015
EPI_ISL_19151078	A/Rheinland-Pfalz/15/2014	EPI_ISL_208324	A/Baden-Wuerttemberg/254/2015
EPI_ISL_19151079	A/Schleswig-Holstein/9/2014	EPI_ISL_208330	A/Berlin/176/2015
EPI_ISL_19151080	A/Bayern/110/2015	EPI_ISL_202564	A/Hamburg/24/2015
EPI_ISL_19151081	A/Brandenburg/29/2015	EPI_ISL_202716	A/Hamburg/25/2015
EPI_ISL_19151082	A/Berlin/78/2015	EPI_ISL_202565	A/Hamburg/26/2015
EPI_ISL_19151083	A/Berlin/113/2015	EPI_ISL_208329	A/Mecklenburg-Vorpommern/20/2015
EPI_ISL_19151084	A/Berlin/97/2015	EPI_ISL_208336	A/Berlin/177/2015
EPI_ISL_19151085	A/Bremen/23/2015	EPI_ISL_208326	A/Mecklenburg-Vorpommern/21/2015
EPI_ISL_19151086	A/Baden-Wuerttemberg/14/2015	EPI_ISL_202717	A/Nordrhein-Westfalen/102/2015
EPI_ISL_19151087	A/Hamburg/23/2015	EPI_ISL_202718	A/Nordrhein-Westfalen/103/2015
EPI_ISL_19151088	A/Hamburg/10/2015	EPI_ISL_203629	A/Nordrhein-Westfalen/105/2015
EPI_ISL_19151089	A/Hessen/37/2015	EPI_ISL_208337	A/Nordrhein-Westfalen/106/2015
EPI_ISL_19151090	A/Nordrhein-Westfalen/77/2015	EPI_ISL_202563	A/Bremen/25/2015
EPI_ISL_19151091	A/Nordrhein-Westfalen/83/2015	EPI_ISL_203626	A/Rheinland-Pfalz/55/2015
EPI_ISL_19151092	A/Nordrhein-Westfalen/93/2015	EPI_ISL_212442	A/Sachsen/102/2015
EPI_ISL_208328	A/Sachsen/104/2015	EPI_ISL_222727	A/Bayern/16/2016
EPI_ISL_203625	A/Sachsen-Anhalt/91/2015	EPI_ISL_222729	A/Bayern/21/2016
EPI_ISL_208332	A/Thueringen/101/2015	EPI_ISL_222731	A/Bayern/23/2016
EPI_ISL_203627	A/Bremen/26/2015	EPI_ISL_222736	A/Bayern/24/2016
EPI_ISL_208338	A/Hamburg/1/2016	EPI_ISL_213449	A/Bayern/30/2016

Table S1. Accession number (Gisaid) of influenza A(H1N1)pdm09 viruses isolated in Germany between 2009 and 2024

EPI_ISL_208334	A/Thueringen/1/2016	EPI_ISL_222613	A/Bayern/36/2016
EPI_ISL_208335	A/Thueringen/2/2016	EPI_ISL_222620	A/Bayern/42/2016
EPI_ISL_208325	A/Bremen/28/2015	EPI_ISL_222655	A/Bayern/45/2016
EPI_ISL_19159374	A/Bremen/27/2015	EPI_ISL_222631	A/Bayern/47/2016
EPI_ISL_19159375	A/Thueringen/103/2015	EPI_ISL_223783	A/Bayern/50/2016
EPI_ISL_19159376	A/Bayern/94/2016	EPI_ISL_223789	A/Bayern/52/2016
EPI_ISL_19159377	A/Bayern/20/2016	EPI_ISL_222724	A/Bayern/54/2016
EPI_ISL_19159378	A/Berlin/1/2016	EPI_ISL_222725	A/Bayern/56/2016
EPI_ISL_19159379	A/Berlin/3/2016	EPI_ISL_222738	A/Bayern/59/2016
EPI_ISL_19159380	A/Rheinland-Pfalz/1/2016	EPI_ISL_222619	A/Bayern/65/2016
EPI_ISL_19159381	A/Schleswig-Holstein/4/2016	EPI_ISL_222633	A/Bayern/66/2016
EPI_ISL_19159386	A/Bremen/10/2015	EPI_ISL_222646	A/Bayern/67/2016
EPI_ISL_19159382	A/Berlin/103/2016	EPI_ISL_222649	A/Bayern/68/2016
EPI_ISL_19159383	A/Berlin/168/2015	EPI_ISL_211850	A/Brandenburg/2/2016
EPI_ISL_19159384	A/Berlin/169/2015	EPI_ISL_211855	A/Brandenburg/3/2016
EPI_ISL_19159385	A/Berlin/172/2015	EPI_ISL_212497	A/Brandenburg/9/2016
EPI_ISL_19159684	A/Bayern/6/2016	EPI_ISL_213308	A/Brandenburg/13/2016
EPI_ISL_19159381	A/Schleswig-Holstein/3/2016	EPI_ISL_222605	A/Brandenburg/15/2016
EPI_ISL_19159386	A/Nordrhein-Westfalen/2/2016	EPI_ISL_213448	A/Brandenburg/17/2016
EPI_ISL_19159382	A/Thueringen/105/2015	EPI_ISL_222733	A/Brandenburg/18/2016
EPI_ISL_222723	A/Thueringen/106/2015	EPI_ISL_223786	A/Brandenburg/22/2016
EPI_ISL_208339	A/Bayern/1/2016	EPI_ISL_208344	A/Berlin/1/2016
EPI_ISL_208347	A/Bayern/2/2016	EPI_ISL_208342	A/Berlin/2/2016
EPI_ISL_208345	A/Bayern/3/2016	EPI_ISL_211848	A/Berlin/4/2016
EPI_ISL_211853	A/Bayern/5/2016	EPI_ISL_208349	A/Berlin/5/2016
EPI_ISL_2120501	A/Bayern/7/2016	EPI_ISL_211854	A/Berlin/6/2016
EPI_ISL_213442	A/Bayern/12/2016	EPI_ISL_208346	A/Berlin/9/2016
EPI_ISL_212495	A/Berlin/13/2016	EPI_ISL_222614	A/Baden-Wuerttemberg/39/2016
EPI_ISL_212498	A/Berlin/15/2016	EPI_ISL_222621	A/Baden-Wuerttemberg/52/2016
EPI_ISL_212503	A/Berlin/25/2016	EPI_ISL_222623	A/Baden-Wuerttemberg/53/2016
EPI_ISL_213437	A/Berlin/30/2016	EPI_ISL_222622	A/Baden-Wuerttemberg/56/2016
EPI_ISL_222609	A/Berlin/35/2016	EPI_ISL_222632	A/Baden-Wuerttemberg/61/2016

Table S1. Accession number (Gisaid) of influenza A(H1N1)pdm09 viruses isolated in Germany between 2009 and 2024

EPI_ISL_213444	A/Berlin/38/2016	EPI_ISL_222647	A/Baden-Wuerttemberg/72/2016
EPI_ISL_222611	A/Berlin/39/2016	EPI_ISL_223782	A/Baden-Wuerttemberg/84/2016
EPI_ISL_213758	A/Berlin/40/2016	EPI_ISL_211851	A/Hamburg/2/2016
EPI_ISL_213445	A/Berlin/41/2016	EPI_ISL_222599	A/Hamburg/3/2016
EPI_ISL_222608	A/Berlin/45/2016	EPI_ISL_212504	A/Hamburg/6/2016
EPI_ISL_218495	A/Berlin/55/2016	EPI_ISL_213438	A/Hessen/3/2016
EPI_ISL_222730	A/Berlin/60/2016	EPI_ISL_222606	A/Hessen/4/2016
EPI_ISL_222616	A/Berlin/71/2016	EPI_ISL_222610	A/Hessen/7/2016
EPI_ISL_222624	A/Berlin/76/2016	EPI_ISL_218493	A/Hessen/8/2016
EPI_ISL_222638	A/Berlin/77/2016	EPI_ISL_218491	A/Hessen/10/2016
EPI_ISL_222726	A/Berlin/84/2016	EPI_ISL_222636	A/Hessen/13/2016
EPI_ISL_222648	A/Berlin/85/2016	EPI_ISL_222659	A/Hessen/14/2016
EPI_ISL_222650	A/Berlin/86/2016	EPI_ISL_222660	A/Hessen/15/2016
EPI_ISL_222651	A/Berlin/87/2016	EPI_ISL_208343	A/Mecklenburg-Vorpommern/1/2016
EPI_ISL_222607	A/Bremen/1/2016	EPI_ISL_222602	A/Mecklenburg-Vorpommern/2/2016
EPI_ISL_222737	A/Bremen/5/2016	EPI_ISL_212505	A/Mecklenburg-Vorpommern/4/2016
EPI_ISL_222735	A/Bremen/6/2016	EPI_ISL_222617	A/Mecklenburg-Vorpommern/8/2016
EPI_ISL_222628	A/Bremen/8/2016	EPI_ISL_208350	A/Nordrhein-Westfalen/1/2016
EPI_ISL_222640	A/Bremen/9/2016	EPI_ISL_211847	A/Nordrhein-Westfalen/3/2016
EPI_ISL_222639	A/Bremen/10/2016	EPI_ISL_212494	A/Nordrhein-Westfalen/4/2016
EPI_ISL_208341	A/Baden-Wuerttemberg/1/2016	EPI_ISL_222600	A/Nordrhein-Westfalen/12/2016
EPI_ISL_212506	A/Baden-Wuerttemberg/7/2016	EPI_ISL_213307	A/Nordrhein-Westfalen/14/2016
EPI_ISL_213447	A/Baden-Wuerttemberg/16/2016	EPI_ISL_222604	A/Nordrhein-Westfalen/15/2016
EPI_ISL_213446	A/Baden-Wuerttemberg/17/2016	EPI_ISL_213309	A/Nordrhein-Westfalen/16/2016
EPI_ISL_222734	A/Baden-Wuerttemberg/25/2016	EPI_ISL_218492	A/Nordrhein-Westfalen/18/2016
EPI_ISL_222612	A/Baden-Wuerttemberg/34/2016	EPI_ISL_212499	A/Nordrhein-Westfalen/19/2016
EPI_ISL_222741	A/Baden-Wuerttemberg/37/2016	EPI_ISL_222728	A/Nordrhein-Westfalen/27/2016
EPI_ISL_222618	A/Nordrhein-Westfalen/34/2016	EPI_ISL_222644	A/Rheinland-Pfalz/35/2016
EPI_ISL_223784	A/Nordrhein-Westfalen/43/2016	EPI_ISL_223788	A/Rheinland-Pfalz/36/2016
EPI_ISL_222625	A/Nordrhein-Westfalen/57/2016	EPI_ISL_223790	A/Saarland/9/2016
EPI_ISL_222601	A/Niedersachsen/2/2016	EPI_ISL_208352	A/Sachsen/1/2016
EPI_ISL_213441	A/Niedersachsen/4/2016	EPI_ISL_208353	A/Sachsen/6/2016

Table S1. Accession number (Gisaid) of influenza A(H1N1)pdm09 viruses isolated in Germany between 2009 and 2024

EPI_ISL_218490	A/Niedersachsen/8/2016	EPI_ISL_211859	A/Thueringen/104/2015
EPI_ISL_213440	A/Niedersachsen/9/2016	EPI_ISL_212000	A/Sachsen/14/2016
EPI_ISL_218494	A/Niedersachsen/12/2016	EPI_ISL_222642	A/Sachsen/54/2016
EPI_ISL_213310	A/Niedersachsen/25/2016	EPI_ISL_222641	A/Sachsen/61/2016
EPI_ISL_222739	A/Niedersachsen/29/2016	EPI_ISL_223785	A/Sachsen/66/2016
EPI_ISL_222615	A/Niedersachsen/35/2016	EPI_ISL_223787	A/Sachsen/68/2016
EPI_ISL_222634	A/Niedersachsen/37/2016	EPI_ISL_222629	A/Sachsen-Anhalt/76/2016
EPI_ISL_222637	A/Niedersachsen/38/2016	EPI_ISL_222645	A/Sachsen-Anhalt/62/2016
EPI_ISL_222653	A/Niedersachsen/39/2016	EPI_ISL_212507	A/Schleswig-Holstein/2/2016
EPI_ISL_222643	A/Niedersachsen/40/2016	EPI_ISL_222627	A/Schleswig-Holstein/16/2016
EPI_ISL_222654	A/Niedersachsen/41/2016	EPI_ISL_208340	A/Thueringen/3/2016
EPI_ISL_222658	A/Niedersachsen/42/2016	EPI_ISL_211852	A/Thueringen/4/2016
EPI_ISL_222630	A/Niedersachsen/54/2016	EPI_ISL_211849	A/Thueringen/5/2016
EPI_ISL_222635	A/Niedersachsen/55/2016	EPI_ISL_213443	A/Thueringen/14/2016
EPI_ISL_222657	A/Niedersachsen/56/2016	EPI_ISL_222732	A/Thueringen/18/2016
EPI_ISL_211846	A/Rheinland-Pfalz/2/2016	EPI_ISL_211856	A/Baden-Wuerttemberg/2/2016
EPI_ISL_208351	A/Rheinland-Pfalz/3/2016	EPI_ISL_211860	A/Sachsen/3/2016
EPI_ISL_212493	A/Rheinland-Pfalz/5/2016	EPI_ISL_211861	A/Sachsen/4/2016
EPI_ISL_212496	A/Rheinland-Pfalz/8/2016	EPI_ISL_211862	A/Sachsen/5/2016
EPI_ISL_212500	A/Rheinland-Pfalz/9/2016	EPI_ISL_253536	A/Nordrhein-Westfalen/26/2017
EPI_ISL_212502	A/Rheinland-Pfalz/11/2016	EPI_ISL_253538	A/Niedersachsen/31/2017
EPI_ISL_213439	A/Rheinland-Pfalz/12/2016	EPI_ISL_253537	A/Rheinland-Pfalz/38/2017
EPI_ISL_213306	A/Rheinland-Pfalz/13/2016	EPI_ISL_253539	A/Sachsen-Anhalt/3/2017
EPI_ISL_222740	A/Rheinland-Pfalz/25/2016	EPI_ISL_19159932	A/Baden-Wuerttemberg/256/2017
EPI_ISL_222626	A/Rheinland-Pfalz/32/2016	EPI_ISL_19159930	A/Baden-Wuerttemberg/16/2018
EPI_ISL_222652	A/Rheinland-Pfalz/33/2016	EPI_ISL_19159931	A/Hessen/45/2018
EPI_ISL_222656	A/Rheinland-Pfalz/34/2016	EPI_ISL_301645	A/Niedersachsen/17/2018
EPI_ISL_19159933	A/Saarland/25/2017	EPI_ISL_301680	A/Rheinland-Pfalz/19/2018
EPI_ISL_288727	A/Rheinland-Pfalz/50/2017	EPI_ISL_301646	A/Baden-Wuerttemberg/47/2018
EPI_ISL_312788	A/Bayern/48/2018	EPI_ISL_311285	A/Baden-Wuerttemberg/56/2018
EPI_ISL_311284	A/Nordrhein-Westfalen/19/2018	EPI_ISL_301647	A/Baden-Wuerttemberg/55/2018
EPI_ISL_222603	A/Sachsen/11/2016	EPI_ISL_292227	A/Nordrhein-Westfalen/135/2017

Table S1. Accession number (Gisaid) of influenza A(H1N1)pdm09 viruses isolated in Germany between 2009 and 2024

EPI_ISL_301644	A/Bayern/7/2018	EPI_ISL_301648	A/Nordrhein-Westfalen/25/2018
EPI_ISL_292228	A/Bayern/96/2017	EPI_ISL_301649	A/Berlin/17/2018
EPI_ISL_292125	A/Saarland/24/2017	EPI_ISL_304442	A/Bayern/17/2018
EPI_ISL_339519	A/Saarland/28/2017	EPI_ISL_304443	A/Berlin/23/2018
EPI_ISL_339520	A/Saarland/29/2017	EPI_ISL_306515	A/Baden-Wuerttemberg/82/2018
EPI_ISL_292126	A/Saarland/26/2017	EPI_ISL_306516	A/Saarland/9/2018
EPI_ISL_339521	A/Saarland/30/2017	EPI_ISL_306517	A/Baden-Wuerttemberg/120/2018
EPI_ISL_339522	A/Saarland/31/2017	EPI_ISL_306678	A/Nordrhein-Westfalen/68/2018
EPI_ISL_293953	A/Nordrhein-Westfalen/138/2017	EPI_ISL_306518	A/Berlin/37/2018
EPI_ISL_293954	A/Baden-Wuerttemberg/257/2017	EPI_ISL_306519	A/Baden-Wuerttemberg/153/2018
EPI_ISL_293955	A/Baden-Wuerttemberg/259/2017	EPI_ISL_306520	A/Baden-Wuerttemberg/154/2018
EPI_ISL_293956	A/Bayern/1/2018	EPI_ISL_306521	A/Nordrhein-Westfalen/65/2018
EPI_ISL_293967	A/Nordrhein-Westfalen/2/2018	EPI_ISL_306522	A/Berlin/55/2018
EPI_ISL_293969	A/Hessen/1/2018	EPI_ISL_311288	A/Baden-Wuerttemberg/156/2018
EPI_ISL_293970	A/Baden-Wuerttemberg/4/2018	EPI_ISL_311289	A/Brandenburg/25/2018
EPI_ISL_293972	A/Bayern/3/2018	EPI_ISL_311306	A/Berlin/56/2018
EPI_ISL_298744	A/Niedersachsen/1/2018	EPI_ISL_311290	A/Niedersachsen/115/2018
EPI_ISL_298747	A/Rheinland-Pfalz/4/2018	EPI_ISL_403461	A/Schleswig-Holstein/5/2019
EPI_ISL_298749	A/Berlin/5/2018	EPI_ISL_378172	A/Sachsen/89/2018
EPI_ISL_301673	A/Baden-Wuerttemberg/86/2018	EPI_ISL_378171	A/Sachsen/88/2018
EPI_ISL_301674	A/Baden-Wuerttemberg/87/2018	EPI_ISL_378162	A/Baden-Wuerttemberg/309/2019
EPI_ISL_301675	A/Baden-Wuerttemberg/88/2018	EPI_ISL_378161	A/Schleswig-Holstein/4/2019
EPI_ISL_301676	A/Baden-Wuerttemberg/89/2018	EPI_ISL_378160	A/Bayern/112/2019
EPI_ISL_301677	A/Baden-Wuerttemberg/90/2018	EPI_ISL_378159	A/Baden-Wuerttemberg/308/2019
EPI_ISL_301678	A/Baden-Wuerttemberg/24/2018	EPI_ISL_378158	A/Baden-Wuerttemberg/53/2019
EPI_ISL_301679	A/Baden-Wuerttemberg/15/2018	EPI_ISL_378157	A/Sachsen/28/2019
EPI_ISL_356688	A/Sachsen/130/2019	EPI_ISL_378156	A/Niedersachsen/194/2019
EPI_ISL_378155	A/Baden-Wuerttemberg/25/2019	EPI_ISL_346525	A/Hessen/40/2019
EPI_ISL_378154	A/Niedersachsen/28/2019	EPI_ISL_346524	A/Niedersachsen/120/2019
EPI_ISL_378153	A/Berlin/7/2019	EPI_ISL_346523	A/Niedersachsen/66/2019
EPI_ISL_356689	A/Thueringen/107/2019	EPI_ISL_346522	A/Bayern/88/2019
EPI_ISL_346521	A/Saarland/6/2019	EPI_ISL_346520	A/Bayern/63/2019

Table S1. Accession number (Gisaid) of influenza A(H1N1)pdm09 viruses isolated in Germany between 2009 and 2024

EPI_ISL_356687	A/Nordrhein-Westfalen/120/2019	EPI_ISL_339515	A/Nordrhein-Westfalen/9/2019
EPI_ISL_356686	A/Bayern/109/2019	EPI_ISL_346519	A/Nordrhein-Westfalen/56/2019
EPI_ISL_355970	A/Hamburg/4/2019	EPI_ISL_346518	A/Niedersachsen/119/2019
EPI_ISL_355968	A/Sachsen-Anhalt/84/2019	EPI_ISL_346517	A/Baden-Wuerttemberg/55/2019
EPI_ISL_355946	A/Hessen/67/2019	EPI_ISL_346516	A/Niedersachsen/40/2019
EPI_ISL_355945	A/Hessen/64/2019	EPI_ISL_343574	A/Berlin/26/2019
EPI_ISL_355944	A/Bayern/107/2019	EPI_ISL_343573	A/Thueringen/46/2019
EPI_ISL_355943	A/Sachsen/111/2019	EPI_ISL_343572	A/Bayern/32/2019
EPI_ISL_355942	A/Niedersachsen/160/2019	EPI_ISL_343571	A/Thueringen/11/2019
EPI_ISL_355941	A/Rheinland-Pfalz/51/2019	EPI_ISL_343570	A/Hessen/17/2019
EPI_ISL_353318	A/Baden-Wuerttemberg/195/2019	EPI_ISL_343569	A/Niedersachsen/26/2019
EPI_ISL_353317	A/Niedersachsen/150/2019	EPI_ISL_343568	A/Rheinland-Pfalz/31/2019
EPI_ISL_353316	A/Bremen/19/2019	EPI_ISL_343567	A/Hessen/16/2019
EPI_ISL_351421	A/Baden-Wuerttemberg/164/2019	EPI_ISL_343566	A/Bremen/7/2019
EPI_ISL_351420	A/Sachsen-Anhalt/43/2019	EPI_ISL_343565	A/Baden-Wuerttemberg/88/2019
EPI_ISL_351419	A/Brandenburg/23/2019	EPI_ISL_343564	A/Baden-Wuerttemberg/21/2019
EPI_ISL_351418	A/Sachsen/125/2019	EPI_ISL_343563	A/Berlin/45/2019
EPI_ISL_351417	A/Sachsen/124/2019	EPI_ISL_343562	A/Baden-Wuerttemberg/15/2019
EPI_ISL_348805	A/Baden-Wuerttemberg/212/2019	EPI_ISL_343561	A/Saarland/3/2019
EPI_ISL_348793	A/Sachsen/81/2019	EPI_ISL_343560	A/Nordrhein-Westfalen/16/2019
EPI_ISL_348792	A/Sachsen-Anhalt/40/2019	EPI_ISL_343559	A/Berlin/11/2019
EPI_ISL_348791	A/Niedersachsen/98/2019	EPI_ISL_343558	A/Rheinland-Pfalz/6/2019
EPI_ISL_348790	A/Bayern/102/2019	EPI_ISL_343557	A/Berlin/10/2019
EPI_ISL_348789	A/Niedersachsen/86/2019	EPI_ISL_343556	A/Nordrhein-Westfalen/12/2019
EPI_ISL_348788	A/Sachsen/109/2019	EPI_ISL_339517	A/Hessen/23/2019
EPI_ISL_348786	A/Saarland/8/2019	EPI_ISL_339516	A/Niedersachsen/11/2019
EPI_ISL_348785	A/Bayern/101/2019	EPI_ISL_482809	A/Nordrhein-Westfalen/37/2020
EPI_ISL_339513	A/Berlin/6/2019	EPI_ISL_645137	A/Berlin/31/2020
EPI_ISL_339512	A/Brandenburg/2/2019	EPI_ISL_491173	A/Hessen/47/2020
EPI_ISL_339510	A/Sachsen-Anhalt/1/2019	EPI_ISL_482847	A/Brandenburg/8/2020
EPI_ISL_339509	A/Niedersachsen/30/2019	EPI_ISL_482810	A/Nordrhein-Westfalen/59/2020
EPI_ISL_339508	A/Brandenburg/1/2019	EPI_ISL_482808	A/Rheinland-Pfalz/18/2020

Table S1. Accession number (Gisaid) of influenza A(H1N1)pdm09 viruses isolated in Germany between 2009 and 2024

EPI_ISL_339506	A/Nordrhein-Westfalen/5/2019	EPI_ISL_481389	A/Hessen/23/2020
EPI_ISL_339505	A/Rheinland-Pfalz/41/2018	EPI_ISL_482807	A/Hessen/30/2020
EPI_ISL_339504	A/Rheinland-Pfalz/16/2019	EPI_ISL_482806	A/Baden-Wuerttemberg/121/2020
EPI_ISL_337000	A/Rheinland-Pfalz/2/2019	EPI_ISL_482805	A/Niedersachsen/52/2020
EPI_ISL_336996	A/Baden-Wuerttemberg/4/2019	EPI_ISL_482804	A/Baden-Wuerttemberg/120/2020
EPI_ISL_336995	A/Bayern/2/2019	EPI_ISL_482803	A/Rheinland-Pfalz/24/2020
EPI_ISL_336994	A/Rheinland-Pfalz/1/2019	EPI_ISL_482802	A/Thueringen/17/2020
EPI_ISL_336993	A/Thueringen/1/2019	EPI_ISL_482801	A/Bayern/43/2020
EPI_ISL_336992	A/Nordrhein-Westfalen/1/2019	EPI_ISL_482800	A/Bayern/26/2020
EPI_ISL_336991	A/Bremen/1/2019	EPI_ISL_482799	A/Sachsen/28/2020
EPI_ISL_336987	A/ Nordrhein-Westfalen/8/2019	EPI_ISL_482798	A/Mecklenburg-Vorpommern/3/2020
EPI_ISL_336985	A/Niedersachsen/1/2019	EPI_ISL_482797	A/Bayern/25/2020
EPI_ISL_336984	A/Bayern/1/2019	EPI_ISL_482796	A/Schleswig-Holstein/4/2020
EPI_ISL_335316	A/Mecklenburg-Vorpommern/5/2018	EPI_ISL_482795	A/Baden-Wuerttemberg/119/2020
EPI_ISL_335310	A/Hamburg/7/2018	EPI_ISL_482794	A/Niedersachsen/49/2020
EPI_ISL_335308	A/Nordrhein-Westfalen/80/2018	EPI_ISL_482793	A/Baden-Wuerttemberg/58/2020
EPI_ISL_335307	A/Hamburg/6/2018	EPI_ISL_482792	A/Bremen/4/2020
EPI_ISL_335306	A/Berlin/58/2018	EPI_ISL_482791	A/Thueringen/13/2020
EPI_ISL_335304	A/Bayern/51/2018	EPI_ISL_482790	A/Thueringen/14/2020
EPI_ISL_335303	A/Bayern/50/2018	EPI_ISL_482789	A/Saarland/4/2020
EPI_ISL_332946	A/Mecklenburg-Vorpommern/4/2018	EPI_ISL_482788	A/Berlin/30/2020
EPI_ISL_332944	A/Baden-Wuerttemberg/163/2018	EPI_ISL_482787	A/Bayern/42/2020
EPI_ISL_332942	A/Thueringen/61/2018	EPI_ISL_482786	A/Brandenburg/10/2020
EPI_ISL_332941	A/Baden-Wuerttemberg/162/2018	EPI_ISL_481430	A/Bremen/13/2020
EPI_ISL_645140	A/Rheinland-Pfalz/35/2020	EPI_ISL_481429	A/Hessen/39/2020
EPI_ISL_645139	A/Berlin/47/2020	EPI_ISL_481428	A/Baden-Wuerttemberg/149/2020
EPI_ISL_645138	A/Hamburg/1/2020	EPI_ISL_481427	A/Nordrhein-Westfalen/77/2020
EPI_ISL_481426	A/Niedersachsen/137/2020	EPI_ISL_481394	A/Bayern/37/2020
EPI_ISL_481425	A/Niedersachsen/98/2020	EPI_ISL_481393	A/Nordrhein-Westfalen/45/2020
EPI_ISL_481424	A/Bayern/49/2020	EPI_ISL_481391	A/Mecklenburg-Vorpommern/4/2020
EPI_ISL_481423	A/Rheinland-Pfalz/32/2020	EPI_ISL_481390	A/Baden-Wuerttemberg/175/2020
EPI_ISL_481422	A/Saarland/9/2020	EPI_ISL_481388	A/Bayern/57/2020

Table S1. Accession number (Gisaid) of influenza A(H1N1)pdm09 viruses isolated in Germany between 2009 and 2024

EPI_ISL_481421	A/Nordrhein-Westfalen/76/2020	EPI_ISL_407178	A/Bremen/1/2020
EPI_ISL_481420	A/Nordrhein-Westfalen/61/2020	EPI_ISL_481387	A/Saarland/6/2020
EPI_ISL_481419	A/Bayern/46/2020	EPI_ISL_481386	A/Hessen/22/2020
EPI_ISL_481418	A/Rheinland-Pfalz/31/2020	EPI_ISL_481385	A/Berlin/67/2020
EPI_ISL_481417	A/Bremen/11/2020	EPI_ISL_481384	A/Berlin/39/2020
EPI_ISL_481416	A/Rheinland-Pfalz/29/2020	EPI_ISL_481383	A/Saarland/5/2020
EPI_ISL_481415	A/Brandenburg/11/2020	EPI_ISL_481382	A/Baden-Wuerttemberg/85/2020
EPI_ISL_481414	A/Baden-Wuerttemberg/139/2020	EPI_ISL_481381	A/Nordrhein-Westfalen/78/2020
EPI_ISL_481413	A/Hessen/43/2020	EPI_ISL_413430	A/Baden-Wuerttemberg/49/2020
EPI_ISL_481412	A/Hessen/42/2020	EPI_ISL_413429	A/Nordrhein-Westfalen/47/2020
EPI_ISL_481411	A/Niedersachsen/91/2020	EPI_ISL_413428	A/Nordrhein-Westfalen/27/2020
EPI_ISL_481410	A/Bayern/54/2020	EPI_ISL_413427	A/Baden-Wuerttemberg/109/2020
EPI_ISL_481409	A/Bayern/55/2020	EPI_ISL_413426	A/Baden-Wuerttemberg/47/2020
EPI_ISL_481408	A/Bayern/53/2020	EPI_ISL_413425	A/Niedersachsen/21/2020
EPI_ISL_481407	A/Baden-Wuerttemberg/176/2020	EPI_ISL_413424	A/Saarland/3/2020
EPI_ISL_481406	A/Niedersachsen/87/2020	EPI_ISL_413423	A/Rheinland-Pfalz/12/2020
EPI_ISL_481405	A/Saarland/7/2020	EPI_ISL_413422	A/Sachsen/14/2020
EPI_ISL_481404	A/Rheinland-Pfalz/21/2020	EPI_ISL_413421	A/Berlin/24/2020
EPI_ISL_481403	A/Berlin/43/2020	EPI_ISL_413420	A/Baden-Wuerttemberg/28/2020
EPI_ISL_481402	A/Schleswig-Holstein/7/2020	EPI_ISL_413419	A/Bayern/19/2020
EPI_ISL_481401	A/Rheinland-Pfalz/36/2020	EPI_ISL_410964	A/Nordrhein-Westfalen/19/2020
EPI_ISL_481400	A/Nordrhein-Westfalen/79/2020	EPI_ISL_410963	A/Baden-Wuerttemberg/22/2020
EPI_ISL_481399	A/Nordrhein-Westfalen/44/2020	EPI_ISL_410962	A/Hessen/18/2020
EPI_ISL_481398	A/Berlin/68/2020	EPI_ISL_410961	A/Niedersachsen/16/2020
EPI_ISL_481397	A/Nordrhein-Westfalen/46/2020	EPI_ISL_410960	A/Berlin/15/2020
EPI_ISL_481396	A/Hessen/41/2020	EPI_ISL_410959	A/Baden-Wuerttemberg/21/2020
EPI_ISL_481395	A/Hessen/24/2020	EPI_ISL_410958	A/Bayern/16/2020
EPI_ISL_410957	A/Bayern/15/2020	EPI_ISL_407182	A/Mecklenburg-Vorpommern/1/2020
EPI_ISL_410956	A/Sachsen/10/2020	EPI_ISL_407181	A/Baden-Wuerttemberg/1/2020
EPI_ISL_410955	A/Bayern/14/2020	EPI_ISL_407180	A/Rheinland-Pfalz/3/2020
EPI_ISL_410954	A/Baden-Wuerttemberg/19/2020	EPI_ISL_407179	A/Brandenburg/2/2020
EPI_ISL_410953	A/Baden-Wuerttemberg/18/2020	EPI_ISL_407177	A/Saarland/1/2020

Table S1. Accession number (Gisaid) of influenza A(H1N1)pdm09 viruses isolated in Germany between 2009 and 2024

EPI_ISL_410952	A/Bayern/9/2020	EPI_ISL_401777	A/Hessen/71/2019
EPI_ISL_410951	A/Nordrhein-Westfalen/14/2020	EPI_ISL_407176	A/Brandenburg/1/2020
EPI_ISL_410950	A/Baden-Wuerttemberg/11/2020	EPI_ISL_407175	A/Nordrhein-Westfalen/2/2020
EPI_ISL_410949	A/Hessen/5/2020	EPI_ISL_407174	A/Thueringen/1/2020
EPI_ISL_410075	A/Niedersachsen/11/2020	EPI_ISL_407173	A/Nordrhein-Westfalen/1/2020
EPI_ISL_410073	A/Baden-Wuerttemberg/5/2020	EPI_ISL_407172	A/Rheinland-Pfalz/61/2019
EPI_ISL_410071	A/Mecklenburg-Vorpommern/2/2020	EPI_ISL_407171	A/Rheinland-Pfalz/60/2019
EPI_ISL_410070	A/Hessen/3/2020	EPI_ISL_407170	A/Schleswig-Holstein/6/2019
EPI_ISL_410068	A/Nordrhein-Westfalen/11/2020	EPI_ISL_407169	A/Schleswig-Holstein/7/2019
EPI_ISL_410066	A/Hessen/2/2020	EPI_ISL_405006	A/Nordrhein-Westfalen/143/2019
EPI_ISL_410065	A/Berlin/12/2020	EPI_ISL_405005	A/Thueringen/125/2019
EPI_ISL_410063	A/Nordrhein-Westfalen/9/2020	EPI_ISL_405004	A/Nordrhein-Westfalen/142/2019
EPI_ISL_410062	A/Niedersachsen/9/2020	EPI_ISL_405003	A/Mecklenburg-Vorpommern/13/2019
EPI_ISL_410060	A/Nordrhein-Westfalen/8/2020	EPI_ISL_405002	A/Hessen/73/2019
EPI_ISL_410059	A/Nordrhein-Westfalen/7/2020	EPI_ISL_405001	A/Mecklenburg-Vorpommern/12/2019
EPI_ISL_410058	A/Nordrhein-Westfalen/6/2020	EPI_ISL_405000	A/Nordrhein-Westfalen/139/2019
EPI_ISL_410057	A/Bayern/7/2020	EPI_ISL_404999	A/Brandenburg/29/2019
EPI_ISL_410056	A/Bayern/5/2020	EPI_ISL_404998	A/Nordrhein-Westfalen/138/2019
EPI_ISL_410055	A/Niedersachsen/4/2020	EPI_ISL_404997	A/Baden-Wuerttemberg/313/2019
EPI_ISL_410054	A/Niedersachsen/2/2020	EPI_ISL_404996	A/Sachsen/152/2019
EPI_ISL_410053	A/Berlin/6/2020	EPI_ISL_404995	A/Sachsen/151/2019
EPI_ISL_407188	A/Sachsen/3/2020	EPI_ISL_404994	A/Nordrhein-Westfalen/136/2019
EPI_ISL_407187	A/Nordrhein-Westfalen/4/2020	EPI_ISL_401781	A/Berlin/59/2019
EPI_ISL_407186	A/Hessen/7/2020	EPI_ISL_401780	A/Berlin/58/2019
EPI_ISL_407185	A/Bayern/1/2020	EPI_ISL_401779	A/Niedersachsen/195/2019
EPI_ISL_407184	A/Thueringen/4/2020	EPI_ISL_401778	A/Berlin/57/2019
EPI_ISL_407183	A/Nordrhein-Westfalen/3/2020	EPI_ISL_17780387	A/Niedersachsen/9/2023
EPI_ISL_2434153	A/Mecklenburg-Vorpommern/1-A/2021	EPI_ISL_17959494	A/Baden-Wuerttemberg/27/2023
EPI_ISL_2434152	A/Mecklenburg-Vorpommern/1/2021	EPI_ISL_17959493	A/Hessen/11/2023
EPI_ISL_10429833	A/Mecklenburg-Vorpommern/2/2022	EPI_ISL_17959492	A/Berlin/19/2023
EPI_ISL_11789577	A/Bremen/3/2022	EPI_ISL_17959491	A/Rheinland-Pfalz/6/2023
EPI_ISL_13503236	A/Rheinland-Pfalz/2/2022	EPI_ISL_17780386	A/Brandenburg/2/2023

Table S1. Accession number (Gisaid) of influenza A(H1N1)pdm09 viruses isolated in Germany between 2009 and 2024

EPI_ISL_12839598	A/Bremen/10/2022	EPI_ISL_17592912	A/Bayern/4/2023
EPI_ISL_12839599	A/Hessen/16/2022	EPI_ISL_17780385	A/Hessen/7/2023
EPI_ISL_13503237	A/Saarland/2/2022	EPI_ISL_17780384	A/Thueringen/5/2023
EPI_ISL_13638360	A/Niedersachsen/12/2022	EPI_ISL_17780383	A/Baden-Wuerttemberg/13/2023
EPI_ISL_14925890	A/Baden-Wuerttemberg/30/2022	EPI_ISL_17780382	A/Niedersachsen/4/2023
EPI_ISL_14934966	A/Nordrhein-Westfalen/31/2022	EPI_ISL_17780308	A/Baden-Wuerttemberg/24/2023
EPI_ISL_227814	A/Niedersachsen/44/2022	EPI_ISL_17780307	A/Berlin/16/2023
EPI_ISL_227814	A/Niedersachsen/45/2022	EPI_ISL_17780306	A/Berlin/8/2023
EPI_ISL_227814	A/Brandenburg/6/2022	EPI_ISL_17780305	A/Nordrhein-Westfalen/9/2023
EPI_ISL_19168065	A/Berlin/270/2022	EPI_ISL_17780304	A/Baden-Wuerttemberg/20/2023
EPI_ISL_19168066	A/Berlin/271/2022	EPI_ISL_17780303	A/Hessen/8/2023
EPI_ISL_19168067	A/Berlin/139/2022	EPI_ISL_17780302	A/Hessen/4/2023
EPI_ISL_19168068	A/Hessen/37/2022	EPI_ISL_17780301	A/Nordrhein-Westfalen/3/2023
EPI_ISL_19168069	A/Rheinland-Pfalz/15/2022	EPI_ISL_17592920	A/Berlin/12/2023
EPI_ISL_18526252	A/Berlin/22/2023	EPI_ISL_17592919	A/Berlin/6/2023
EPI_ISL_18526251	A/Niedersachsen/21/2023	EPI_ISL_17592918	A/Nordrhein-Westfalen/8/2023
EPI_ISL_18526250	A/Berlin/14/2023	EPI_ISL_17592917	A/Sachsen/3/2023
EPI_ISL_18514884	A/Berlin/26/2023	EPI_ISL_17592916	A/Berlin/2/2023
EPI_ISL_18412346	A/Nordrhein-Westfalen/10/2023	EPI_ISL_17592915	A/Bayern/7/2023
EPI_ISL_18412345	A/Sachsen-Anhalt/8/2023	EPI_ISL_17592914	A/Saarland/1/2023
EPI_ISL_18412344	A/Baden-Wuerttemberg/27/2023	EPI_ISL_17592913	A/Berlin/11/2023
EPI_ISL_17959500	A/Schleswig-Holstein/2/2023	EPI_ISL_17197521	A/Schweiz/1/2023
EPI_ISL_17959499	A/Berlin/21/2023	EPI_ISL_16548602	A/Berlin/220/2022
EPI_ISL_17959498	A/Rheinland-Pfalz/5/2023	EPI_ISL_17197520	A/Baden-Wuerttemberg/158/2022
EPI_ISL_17959497	A/Hessen/13/2023	EPI_ISL_17037583	A/Nordrhein-Westfalen/2/2023
EPI_ISL_17959496	A/Hessen/12/2023	EPI_ISL_17037582	A/Nordrhein-Westfalen/6/2023
EPI_ISL_17959495	A/Berlin/20/2023	EPI_ISL_17037581	A/Nordrhein-Westfalen/1/2023
EPI_ISL_17037580	A/Mecklenburg-Vorpommern/1/2023	EPI_ISL_17037548	A/Nordrhein-Westfalen/58/2022
EPI_ISL_17037579	A/Bayern/1/2023	EPI_ISL_16548605	A/Hamburg/4/2022
EPI_ISL_17037578	A/Thueringen/4/2023	EPI_ISL_16548604	A/Baden-Wuerttemberg/108/2022
EPI_ISL_17037577	A/Baden-Wuerttemberg/1/2023	EPI_ISL_16548603	A/Nordrhein-Westfalen/90/2022
EPI_ISL_17037576	A/Bayern/48/2022	EPI_ISL_16548601	A/Thueringen/46/2022

Table S1. Accession number (Gisaid) of influenza A(H1N1)pdm09 viruses isolated in Germany between 2009 and 2024

EPI_ISL_17037575	A/Berlin/228/2022	EPI_ISL_16103551	A/Nordrhein-Westfalen/39/2022
EPI_ISL_17037574	A/Baden-Wuerttemberg/96/2022	EPI_ISL_16548599	A/Thueringen/81/2022
EPI_ISL_17037573	A/Baden-Wuerttemberg/95/2022	EPI_ISL_16103552	A/Schleswig-Holstein/19/2022
EPI_ISL_17037572	A/Berlin/211/2022	EPI_ISL_18993828	A/Nordrhein-Westfalen/67/2024
EPI_ISL_17037571	A/Nordrhein-Westfalen/88/2022	EPI_ISL_19002011	A/Schleswig-Holstein/44/2024
EPI_ISL_17037570	A/Berlin/209/2022	EPI_ISL_19002010	A/Schleswig-Holstein/43/2024
EPI_ISL_17037569	A/Nordrhein-Westfalen/85/2022	EPI_ISL_19002009	A/Schleswig-Holstein/42/2024
EPI_ISL_17037568	A/Nordrhein-Westfalen/87/2022	EPI_ISL_19002008	A/Schleswig-Holstein/41/2024
EPI_ISL_17037567	A/Berlin/227/2022	EPI_ISL_19002007	A/Schleswig-Holstein/28/2024
EPI_ISL_17037566	A/Sachsen/30/2022	EPI_ISL_19002006	A/Schleswig-Holstein/40/2024
EPI_ISL_17037565	A/Baden-Wuerttemberg/94/2022	EPI_ISL_19002005	A/Schleswig-Holstein/39/2024
EPI_ISL_17037564	A/Rheinland-Pfalz/36/2022	EPI_ISL_19002004	A/Schleswig-Holstein/24/2024
EPI_ISL_17037563	A/Thueringen/73/2022	EPI_ISL_19002003	A/Thueringen/63/2024
EPI_ISL_17037562	A/Baden-Wuerttemberg/92/2022	EPI_ISL_19002002	A/Nordrhein-Westfalen/72/2024
EPI_ISL_17037561	A/Niedersachsen/106/2022	EPI_ISL_19002001	A/Nordrhein-Westfalen/71/2024
EPI_ISL_17037560	A/Berlin/203/2022	EPI_ISL_19002000	A/Hessen/80/2024
EPI_ISL_17037559	A/Berlin/202/2022	EPI_ISL_19001999	A/Hessen/79/2024
EPI_ISL_17037558	A/Berlin/201/2022	EPI_ISL_19001998	A/Bremen/21/2024
EPI_ISL_17037557	A/Berlin/198/2022	EPI_ISL_19001997	A/Bremen/20/2024
EPI_ISL_17037556	A/Berlin/196/2022	EPI_ISL_19001996	A/Hessen/78/2024
EPI_ISL_17037555	A/Berlin/230/2022	EPI_ISL_19001995	A/Rheinland-Pfalz/31/2024
EPI_ISL_17037554	A/Nordrhein-Westfalen/76/2022	EPI_ISL_19001994	A/Baden-Wuerttemberg/136/2024
EPI_ISL_17037553	A/Berlin/181/2022	EPI_ISL_19001993	A/Hessen/77/2024
EPI_ISL_17037552	A/Berlin/178/2022	EPI_ISL_19001992	A/Nordrhein-Westfalen/70/2024
EPI_ISL_17037551	A/Nordrhein-Westfalen/59/2022	EPI_ISL_19001991	A/Nordrhein-Westfalen/69/2024
EPI_ISL_17037550	A/Berlin/226/2022	EPI_ISL_19001990	A/Saarland/15/2024
EPI_ISL_17037549	A/Berlin/224/2022	EPI_ISL_19001989	A/Nordrhein-Westfalen/68/2024
EPI_ISL_19001988	A/Schleswig-Holstein/23/2024	EPI_ISL_18993832	A/Bayern/61/2024
EPI_ISL_19001987	A/Hessen/76/2024	EPI_ISL_18993831	A/Hessen/69/2024
EPI_ISL_19001986	A/Baden-Wuerttemberg/135/2024	EPI_ISL_18993830	A/Hessen/68/2024
EPI_ISL_19001985	A/Niedersachsen/75/2024	EPI_ISL_18993829	A/Hessen/67/2024
EPI_ISL_19001984	A/Berlin/30/2024	EPI_ISL_18993827	A/Bayern/60/2024

Table S1. Accession number (Gisaid) of influenza A(H1N1)pdm09 viruses isolated in Germany between 2009 and 2024

EPI_ISL_19001983	A/Bayern/69/2024	EPI_ISL_18969203	A/Hessen/59/2024
EPI_ISL_19001982	A/Bayern/68/2024	EPI_ISL_18993826	A/Bayern/59/2024
EPI_ISL_19001981	A/Bayern/67/2024	EPI_ISL_18993825	A/Bremen/19/2024
EPI_ISL_19001980	A/Bayern/66/2024	EPI_ISL_18993824	A/Schleswig-Holstein/37/2024
EPI_ISL_19001979	A/Hessen/75/2024	EPI_ISL_18993823	A/Niedersachsen/96/2024
EPI_ISL_19001978	A/Hessen/74/2024	EPI_ISL_18993822	A/Baden-Wuerttemberg/131/2024
EPI_ISL_19001977	A/Bayern/65/2024	EPI_ISL_18993821	A/Hessen/66/2024
EPI_ISL_19001976	A/Bayern/64/2024	EPI_ISL_18993820	A/Bayern/58/2024
EPI_ISL_19001975	A/Bayern/63/2024	EPI_ISL_18993819	A/Bayern/57/2024
EPI_ISL_19001974	A/Mecklenburg-Vorpommern/15/2024	EPI_ISL_18993818	A/Bayern/56/2024
EPI_ISL_19001973	A/Mecklenburg-Vorpommern/11/2024	EPI_ISL_18993817	A/Hessen/65/2024
EPI_ISL_18993848	A/Schleswig-Holstein/38/2024	EPI_ISL_18993816	A/Hessen/64/2024
EPI_ISL_18993847	A/Brandenburg/27/2024	EPI_ISL_18993815	A/Brandenburg/17/2024
EPI_ISL_18993846	A/Niedersachsen/98/2024	EPI_ISL_18993814	A/Brandenburg/26/2024
EPI_ISL_18993845	A/Niedersachsen/97/2024	EPI_ISL_18993813	A/Thueringen/61/2024
EPI_ISL_18993844	A/Bayern/62/2024	EPI_ISL_18993812	A/Baden-Wuerttemberg/130/2024
EPI_ISL_18993843	A/Baden-Wuerttemberg/134/2024	EPI_ISL_18993811	A/Bremen/15/2024
EPI_ISL_18993842	A/Baden-Wuerttemberg/133/2024	EPI_ISL_18993810	A/Hessen/63/2024
EPI_ISL_18993841	A/Rheinland-Pfalz/30/2024	EPI_ISL_18993809	A/Rheinland-Pfalz/28/2024
EPI_ISL_18993840	A/Saarland/14/2024	EPI_ISL_18993808	A/Niedersachsen/49/2024
EPI_ISL_18993839	A/Hessen/73/2024	EPI_ISL_18993807	A/Niedersachsen/95/2024
EPI_ISL_18993838	A/Hessen/72/2024	EPI_ISL_18993806	A/Mecklenburg-Vorpommern/14/2024
EPI_ISL_18993837	A/Rheinland-Pfalz/29/2024	EPI_ISL_18983414	A/Bremen/18/2024
EPI_ISL_18993836	A/Baden-Wuerttemberg/132/2024	EPI_ISL_18983413	A/Sachsen-Anhalt/26/2024
EPI_ISL_18993835	A/Thueringen/62/2024	EPI_ISL_18983412	A/Thueringen/57/2024
EPI_ISL_18993834	A/Hessen/71/2024	EPI_ISL_18983411	A/Thueringen/56/2024
EPI_ISL_18993833	A/Hessen/70/2024	EPI_ISL_18983410	A/Thueringen/55/2024
EPI_ISL_18983409	A/Rheinland-Pfalz/16/2024	EPI_ISL_18976589	A/Niedersachsen/79/2024
EPI_ISL_18983408	A/Rheinland-Pfalz/15/2024	EPI_ISL_18976588	A/Nordrhein-Westfalen/58/2024
EPI_ISL_18983407	A/Thueringen/54/2024	EPI_ISL_18976587	A/Bayern/48/2024
EPI_ISL_18983406	A/Baden-Wuerttemberg/122/2024	EPI_ISL_18969204	A/Nordrhein-Westfalen/55/2024
EPI_ISL_18983405	A/Hessen/62/2024	EPI_ISL_18969202	A/Hessen/58/2024

Table S1. Accession number (Gisaid) of influenza A(H1N1)pdm09 viruses isolated in Germany between 2009 and 2024

EPI_ISL_18983404	A/Bayern/54/2024	EPI_ISL_18952709	A/Nordrhein-Westfalen/49/2024
EPI_ISL_18983403	A/Mecklenburg-Vorpommern/10/2024	EPI_ISL_18969201	A/Rheinland-Pfalz/27/2024
EPI_ISL_18983402	A/Baden-Wuerttemberg/65/2024	EPI_ISL_18969200	A/Rheinland-Pfalz/26/2024
EPI_ISL_18983401	A/Baden-Wuerttemberg/121/2024	EPI_ISL_18969199	A/Hessen/57/2024
EPI_ISL_18983400	A/Thueringen/27/2024	EPI_ISL_18969198	A/Baden-Wuerttemberg/111/2024
EPI_ISL_18983399	A/Bayern/53/2024	EPI_ISL_18969197	A/Bayern/21/2024
EPI_ISL_18983398	A/Nordrhein-Westfalen/41/2024	EPI_ISL_18969196	A/Niedersachsen/78/2024
EPI_ISL_18983397	A/Bayern/52/2024	EPI_ISL_18969195	A/Baden-Wuerttemberg/110/2024
EPI_ISL_18983396	A/Berlin/29/2024	EPI_ISL_18969194	A/Rheinland-Pfalz/25/2024
EPI_ISL_18983395	A/Hessen/34/2024	EPI_ISL_18969193	A/Hessen/56/2024
EPI_ISL_18983394	A/Hessen/46/2024	EPI_ISL_18969192	A/Baden-Wuerttemberg/109/2024
EPI_ISL_18983393	A/Nordrhein-Westfalen/62/2024	EPI_ISL_18969191	A/Baden-Wuerttemberg/108/2024
EPI_ISL_18983392	A/Nordrhein-Westfalen/39/2024	EPI_ISL_18969190	A/Hessen/55/2024
EPI_ISL_18983391	A/Bayern/51/2024	EPI_ISL_18969189	A/Hessen/54/2024
EPI_ISL_18983390	A/Bayern/39/2024	EPI_ISL_18969188	A/Hessen/22/2024
EPI_ISL_18983389	A/Nordrhein-Westfalen/61/2024	EPI_ISL_18969187	A/Hessen/53/2024
EPI_ISL_18983387	A/Schleswig-Holstein/36/2024	EPI_ISL_18969186	A/Nordrhein-Westfalen/54/2024
EPI_ISL_18983386	A/Schleswig-Holstein/35/2024	EPI_ISL_18969185	A/Nordrhein-Westfalen/53/2024
EPI_ISL_18983385	A/Schleswig-Holstein/34/2024	EPI_ISL_18969184	A/Mecklenburg-Vorpommern/12/2024
EPI_ISL_18983384	A/Schleswig-Holstein/33/2024	EPI_ISL_18969183	A/Baden-Wuerttemberg/107/2024
EPI_ISL_18983383	A/Schleswig-Holstein/32/2024	EPI_ISL_18969182	A/Baden-Wuerttemberg/106/2024
EPI_ISL_18983382	A/Schleswig-Holstein/31/2024	EPI_ISL_18969181	A/Bayern/45/2024
EPI_ISL_18983381	A/Baden-Wuerttemberg/84/2024	EPI_ISL_18969180	A/Bayern/44/2024
EPI_ISL_18983380	A/Baden-Wuerttemberg/112/2024	EPI_ISL_18969179	A/Bayern/43/2024
EPI_ISL_18976592	A/Nordrhein-Westfalen/60/2024	EPI_ISL_18969178	A/Baden-Wuerttemberg/105/2024
EPI_ISL_18976591	A/Nordrhein-Westfalen/59/2024	EPI_ISL_18969177	A/Saarland/13/2024
EPI_ISL_18976590	A/Bayern/49/2024	EPI_ISL_18969176	A/Baden-Wuerttemberg/104/2024
EPI_ISL_18969175	A/Baden-Wuerttemberg/103/2024	EPI_ISL_18952714	A/Nordrhein-Westfalen/51/2024
EPI_ISL_18969174	A/Hessen/52/2024	EPI_ISL_18952713	A/Thueringen/41/2024
EPI_ISL_18969173	A/Hessen/51/2024	EPI_ISL_18952712	A/Rheinland-Pfalz/24/2024
EPI_ISL_18969172	A/Hessen/50/2024	EPI_ISL_18952711	A/Rheinland-Pfalz/23/2024
EPI_ISL_18969171	A/Hessen/49/2024	EPI_ISL_18952708	A/Baden-Wuerttemberg/52/2024

Table S1. Accession number (Gisaid) of influenza A(H1N1)pdm09 viruses isolated in Germany between 2009 and 2024

EPI_ISL_18969170	A/Nordrhein-Westfalen/52/2024	EPI_ISL_18941643	A/Thueringen/40/2024
EPI_ISL_18969169	A/Thueringen/44/2024	EPI_ISL_18952707	A/Berlin/25/2024
EPI_ISL_18969168	A/Bayern/42/2024	EPI_ISL_18952706	A/Brandenburg/23/2024
EPI_ISL_18969167	A/Bremen/12/2024	EPI_ISL_18952705	A/Baden-Wuerttemberg/91/2024
EPI_ISL_18969166	A/Schleswig-Holstein/29/2024	EPI_ISL_18952704	A/Niedersachsen/76/2024
EPI_ISL_18969165	A/Baden-Wuerttemberg/102/2024	EPI_ISL_18952703	A/Niedersachsen/35/2024
EPI_ISL_18969164	A/Brandenburg/25/2024	EPI_ISL_18952702	A/Niedersachsen/34/2024
EPI_ISL_18969163	A/Baden-Wuerttemberg/101/2024	EPI_ISL_18952701	A/Berlin/18/2024
EPI_ISL_18969162	A/Thueringen/43/2024	EPI_ISL_18952700	A/Rheinland-Pfalz/22/2024
EPI_ISL_18961387	A/Baden-Wuerttemberg/100/2024	EPI_ISL_18952699	A/Sachsen/9/2024
EPI_ISL_18961386	A/Niedersachsen/1/2024	EPI_ISL_18952698	A/Hessen/48/2024
EPI_ISL_18961385	A/Brandenburg/24/2024	EPI_ISL_18952697	A/Hessen/47/2024
EPI_ISL_18961384	A/Bayern/1/2024	EPI_ISL_18952696	A/Baden-Wuerttemberg/90/2024
EPI_ISL_18961383	A/Bayern/41/2024	EPI_ISL_18952695	A/Baden-Wuerttemberg/89/2024
EPI_ISL_18961382	A/Thueringen/42/2024	EPI_ISL_18952694	A/Baden-Wuerttemberg/30/2024
EPI_ISL_18961381	A/Bayern/18/2023	EPI_ISL_18952693	A/Baden-Wuerttemberg/88/2024
EPI_ISL_18961380	A/Bayern/17/2023	EPI_ISL_18952692	A/Hessen/16/2024
EPI_ISL_18961379	A/Niedersachsen/26/2023	EPI_ISL_18952691	A/Baden-Wuerttemberg/87/2024
EPI_ISL_18961378	A/Hessen/47/2023	EPI_ISL_18952690	A/Mecklenburg-Vorpommern/7/2024
EPI_ISL_18961377	A/Bayern/21/2023	EPI_ISL_18952689	A/Nordrhein-Westfalen/48/2024
EPI_ISL_18961376	A/Hessen/46/2023	EPI_ISL_18952688	A/Rheinland-Pfalz/21/2024
EPI_ISL_18961375	A/Hessen/45/2023	EPI_ISL_18952687	A/Bayern/20/2024
EPI_ISL_18961374	A/Baden-Wuerttemberg/54/2023	EPI_ISL_18952686	A/Schleswig-Holstein/7/2024
EPI_ISL_18961373	A/Baden-Wuerttemberg/53/2023	EPI_ISL_18952685	A/Nordrhein-Westfalen/21/2024
EPI_ISL_18961372	A/Baden-Wuerttemberg/34/2023	EPI_ISL_18952684	A/Brandenburg/22/2024
EPI_ISL_18961371	A/Hessen/34/2023	EPI_ISL_18952683	A/Baden-Wuerttemberg/86/2024
EPI_ISL_18952715	A/Niedersachsen/36/2024	EPI_ISL_18952682	A/Baden-Wuerttemberg/85/2024
EPI_ISL_18952680	A/Nordrhein-Westfalen/20/2024	EPI_ISL_18941647	A/Hessen/41/2024
EPI_ISL_18952679	A/Saarland/9/2024	EPI_ISL_18941646	A/Niedersachsen/65/2024
EPI_ISL_18942341	A/Nordrhein-Westfalen/18/2024	EPI_ISL_18941645	A/Baden-Wuerttemberg/76/2024
EPI_ISL_18942340	A/Niedersachsen/67/2024	EPI_ISL_18941644	A/Rheinland-Pfalz/18/2024
EPI_ISL_18942339	A/Baden-Wuerttemberg/82/2024	EPI_ISL_18942338	A/Rheinland-Pfalz/5/2024

Table S1. Accession number (Gisaid) of influenza A(H1N1)pdm09 viruses isolated in Germany between 2009 and 2024

EPI_ISL_18941650	A/Nordrhein-Westfalen/46/2024	*EPI_ISL_18941642*	*A/Baden-Wuerttemberg/75/2024*
EPI_ISL_18942337	A/Nordrhein-Westfalen/47/2024	EPI_ISL_19179543	A/Baden-Wuerttemberg/75/2024*(cell-cultured)
EPI_ISL_18942336	A/Thueringen/6/2024	EPI_ISL_18941641	A/Hessen/40/2024
EPI_ISL_18942334	A/Schleswig-Holstein/27/2024	EPI_ISL_18941640	A/Baden-Wuerttemberg/74/2024
EPI_ISL_18942333	A/Schleswig-Holstein/26/2024	EPI_ISL_18941639	A/Hessen/39/2024
EPI_ISL_18941825	A/Hessen/35/2024	EPI_ISL_18941638	A/Hessen/38/2024
EPI_ISL_18941740	A/Hessen/45/2024	EPI_ISL_18941637	A/Hessen/37/2024
EPI_ISL_18941667	A/Hessen/44/2024	EPI_ISL_18941636	A/Hessen/36/2024
EPI_ISL_18941666	A/Rheinland-Pfalz/20/2024	EPI_ISL_18941635	A/Bayern/35/2024
EPI_ISL_18941665	A/Rheinland-Pfalz/19/2024	EPI_ISL_18941634	A/Bayern/34/2024
EPI_ISL_18941664	A/Brandenburg/21/2024	EPI_ISL_18941633	A/Rheinland-Pfalz/17/2024
EPI_ISL_18941663	A/Brandenburg/20/2024	EPI_ISL_18941632	A/Sachsen/8/2024
EPI_ISL_18941662	A/Baden-Wuerttemberg/81/2024	EPI_ISL_18941631	A/Thueringen/39/2024
EPI_ISL_18941661	A/Baden-Wuerttemberg/80/2024	EPI_ISL_18941630	A/Nordrhein-Westfalen/45/2024
EPI_ISL_18941660	A/Baden-Wuerttemberg/79/2024	EPI_ISL_18941629	A/Thueringen/38/2024
EPI_ISL_18941659	A/Hessen/43/2024	EPI_ISL_18941628	A/Niedersachsen/64/2024
EPI_ISL_18941658	A/Bayern/38/2024	EPI_ISL_18941627	A/Niedersachsen/63/2024
EPI_ISL_18941657	A/Sachsen/18/2024	EPI_ISL_18941626	A/Bayern/33/2024
EPI_ISL_18941656	A/Sachsen/17/2024	EPI_ISL_18941625	A/Thueringen/37/2024
EPI_ISL_18941655	A/Bayern/37/2024	EPI_ISL_18941624	A/Bayern/32/2024
EPI_ISL_18941654	A/Bayern/36/2024	EPI_ISL_18941623	A/Brandenburg/19/2024
EPI_ISL_18941653	A/Niedersachsen/66/2024	EPI_ISL_18941622	A/Brandenburg/18/2024
EPI_ISL_18941652	A/Baden-Wuerttemberg/78/2024	EPI_ISL_18941621	A/Thueringen/36/2024
EPI_ISL_18941651	A/Baden-Wuerttemberg/77/2024	EPI_ISL_18926982	A/Nordrhein-Westfalen/36/2024
EPI_ISL_18900973	A/Baden-Wuerttemberg/51/2024	EPI_ISL_18926981	A/Niedersachsen/62/2024
EPI_ISL_18941649	A/Hessen/13/2024	EPI_ISL_18926980	A/Thueringen/26/2024
EPI_ISL_18941648	A/Hessen/42/2024	EPI_ISL_18926979	A/Nordrhein-Westfalen/29/2024
EPI_ISL_18926977	A/Schleswig-Holstein/21/2024	EPI_ISL_18926978	A/Schleswig-Holstein/22/2024
EPI_ISL_18926976	A/Schleswig-Holstein/20/2024	EPI_ISL_18900976	A/Thueringen/11/2024
EPI_ISL_18926975	A/Schleswig-Holstein/19/2024	EPI_ISL_18900975	A/Hessen/24/2024
EPI_ISL_18926974	A/Schleswig-Holstein/18/2024	EPI_ISL_18900974	A/Baden-Wuerttemberg/8/2024
EPI_ISL_18926973	A/Schleswig-Holstein/17/2024	EPI_ISL_18900972	A/Baden-Wuerttemberg/50/2024

Table S1. Accession number (Gisaid) of influenza A(H1N1)pdm09 viruses isolated in Germany between 2009 and 2024

EPI_ISL_18926972	A/Nordrhein-Westfalen/35/2024	EPI_ISL_18887039	A/Rheinland-Pfalz/9/2024
EPI_ISL_18926971	A/Rheinland-Pfalz/14/2024	EPI_ISL_18900971	A/Sachsen-Anhalt/16/2024
EPI_ISL_18926970	A/Nordrhein-Westfalen/34/2024	EPI_ISL_18900970	A/Niedersachsen/38/2024
EPI_ISL_18926969	A/Nordrhein-Westfalen/33/2024	EPI_ISL_18900969	A/Niedersachsen/37/2024
EPI_ISL_18926968	A/Hessen/10/2024	EPI_ISL_18900968	A/Brandenburg/16/2024
EPI_ISL_18926967	A/Bremen/8/2024	EPI_ISL_18900967	A/Baden-Wuerttemberg/49/2024
EPI_ISL_18926966	A/Bremen/6/2024	EPI_ISL_18900966	A/Brandenburg/15/2024
EPI_ISL_18926965	A/Baden-Wuerttemberg/64/2024	EPI_ISL_18900965	A/Brandenburg/14/2024
EPI_ISL_18926964	A/Niedersachsen/61/2024	EPI_ISL_18900964	A/Schleswig-Holstein/2/2024
EPI_ISL_18926963	A/Bayern/9/2024	EPI_ISL_18900963	A/Nordrhein-Westfalen/31/2024
EPI_ISL_18926962	A/Niedersachsen/60/2024	EPI_ISL_18900962	A/Hessen/23/2024
EPI_ISL_18926961	A/Niedersachsen/59/2024	EPI_ISL_18900961	A/Bayern/25/2024
EPI_ISL_18926960	A/Bayern/30/2024	EPI_ISL_18900960	A/Sachsen/6/2024
EPI_ISL_18926959	A/Baden-Wuerttemberg/10/2024	EPI_ISL_18900959	A/Saarland/6/2024
EPI_ISL_18926958	A/Bayern/29/2024	EPI_ISL_18900958	A/Baden-Wuerttemberg/48/2024
EPI_ISL_18926957	A/Hessen/30/2024	EPI_ISL_18900957	A/Mecklenburg-Vorpommern/4/2024
EPI_ISL_18926956	A/Bayern/28/2024	EPI_ISL_18900956	A/Berlin/9/2024
EPI_ISL_18926955	A/Hessen/29/2024	EPI_ISL_18900955	A/Baden-Wuerttemberg/47/2024
EPI_ISL_18926954	A/Hessen/28/2024	EPI_ISL_18900954	A/Bremen/3/2024
EPI_ISL_18926953	A/Bayern/27/2024	EPI_ISL_18900953	A/Baden-Wuerttemberg/46/2024
EPI_ISL_18926952	A/Niedersachsen/20/2024	EPI_ISL_18900952	A/Hessen/7/2024
EPI_ISL_18926951	A/Nordrhein-Westfalen/19/2024	EPI_ISL_18900951	A/Bayern/24/2024
EPI_ISL_18900981	A/Berlin/13/2024	EPI_ISL_18900950	A/Brandenburg/7/2024
EPI_ISL_18514885	*A/Niedersachsen/22/2023*	EPI_ISL_18900949	A/Baden-Wuerttemberg/45/2024
EPI_ISL_19179546	A/Niedersachsen/22/2023*(cell-cultured)	EPI_ISL_18900948	A/Thueringen/4/2024
EPI_ISL_18900979	A/Rheinland-Pfalz/13/2024	EPI_ISL_18900947	A/Nordrhein-Westfalen/30/2024
EPI_ISL_18900978	A/Hessen/25/2024	EPI_ISL_18900946	A/Bayern/23/2024
EPI_ISL_18900977	A/Bremen/13/2024	EPI_ISL_18900945	A/Bayern/22/2024
EPI_ISL_18900944	A/Niedersachsen/9/2024	EPI_ISL_18887042	A/Schleswig-Holstein/8/2024
EPI_ISL_18887073	A/Baden-Wuerttemberg/43/2024	EPI_ISL_18887041	A/Rheinland-Pfalz/10/2024
EPI_ISL_18887072	A/Baden-Wuerttemberg/42/2024	EPI_ISL_18887040	A/Rheinland-Pfalz/3/2024
EPI_ISL_18887071	A/Hessen/21/2024	EPI_ISL_18887038	A/Bayern/18/2024

Table S1. Accession number (Gisaid) of influenza A(H1N1)pdm09 viruses isolated in Germany between 2009 and 2024

EPI_ISL_18887070	A/Bayern/19/2024	EPI_ISL_18869445	A/Brandenburg/1/2024
EPI_ISL_18887069	A/Baden-Wuerttemberg/41/2024	EPI_ISL_18887037	A/Bayern/17/2024
EPI_ISL_18887068	A/Rheinland-Pfalz/11/2024	EPI_ISL_18885546	A/Nordrhein-Westfalen/22/2024
EPI_ISL_18887067	A/Baden-Wuerttemberg/7/2024	EPI_ISL_18882014	A/Bayern/3/2024
EPI_ISL_18887066	A/Nordrhein-Westfalen/27/2024	EPI_ISL_18882013	A/Bayern/16/2024
EPI_ISL_18887065	A/Baden-Wuerttemberg/40/2024	EPI_ISL_18882012	A/Bayern/15/2024
EPI_ISL_18887064	A/Hessen/20/2024	EPI_ISL_18882011	A/Bayern/14/2024
EPI_ISL_18887063	A/Baden-Wuerttemberg/39/2024	EPI_ISL_18882010	A/Rheinland-Pfalz/2/2024
EPI_ISL_18887062	A/Hessen/19/2024	EPI_ISL_18882009	A/Hessen/15/2024
EPI_ISL_18887061	A/Nordrhein-Westfalen/26/2024	EPI_ISL_18882008	A/Bayern/2/2024
EPI_ISL_18887060	A/Hessen/18/2024	EPI_ISL_18882007	A/Bayern/13/2024
EPI_ISL_18887059	A/Nordrhein-Westfalen/8/2024	EPI_ISL_18882006	A/Bremen/11/2024
EPI_ISL_18887058	A/Nordrhein-Westfalen/25/2024	EPI_ISL_18882005	A/Bremen/9/2024
EPI_ISL_18887057	A/Nordrhein-Westfalen/24/2024	EPI_ISL_18882004	A/Baden-Wuerttemberg/27/2024
EPI_ISL_18887056	A/Nordrhein-Westfalen/23/2024	EPI_ISL_18882003	A/Brandenburg/12/2024
EPI_ISL_18887055	A/Brandenburg/6/2024	EPI_ISL_18882002	A/Hessen/14/2024
EPI_ISL_18887054	A/Hessen/17/2024	EPI_ISL_18882001	A/Bayern/12/2024
EPI_ISL_18887053	A/Berlin/20/2024	EPI_ISL_18882000	A/Thueringen/9/2024
EPI_ISL_18887052	A/Berlin/7/2024	EPI_ISL_18881999	A/Baden-Wuerttemberg/3/2024
EPI_ISL_18887051	A/Schleswig-Holstein/10/2023	EPI_ISL_18881998	A/Brandenburg/11/2024
EPI_ISL_18887050	A/Schleswig-Holstein/15/2024	EPI_ISL_18881997	A/Nordrhein-Westfalen/7/2024
EPI_ISL_18887049	A/Schleswig-Holstein/14/2024	EPI_ISL_18881996	A/Nordrhein-Westfalen/5/2024
EPI_ISL_18887048	A/Schleswig-Holstein/13/2024	EPI_ISL_18881995	A/Baden-Wuerttemberg/26/2024
EPI_ISL_18887047	A/Schleswig-Holstein/9/2023	EPI_ISL_18881994	A/Brandenburg/14/2023
EPI_ISL_18887046	A/Schleswig-Holstein/12/2024	EPI_ISL_18881993	A/Brandenburg/13/2023
EPI_ISL_18887045	A/Schleswig-Holstein/11/2024	EPI_ISL_18881992	A/Nordrhein-Westfalen/3/2024
EPI_ISL_18887044	A/Schleswig-Holstein/10/2024	EPI_ISL_18881991	A/Saarland/1/2024
EPI_ISL_18887043	A/Schleswig-Holstein/9/2024	EPI_ISL_18881990	A/Sachsen/2/2024
EPI_ISL_18881989	A/Baden-Wuerttemberg/50/2023	EPI_ISL_18869448	A/Nordrhein-Westfalen/15/2024
EPI_ISL_18881988	A/Baden-Wuerttemberg/49/2023	EPI_ISL_18869447	A/Rheinland-Pfalz/1/2024
EPI_ISL_18881987	A/Baden-Wuerttemberg/48/2023	EPI_ISL_18869446	A/Berlin/2/2024
EPI_ISL_18881986	A/Brandenburg/12/2023	EPI_ISL_18869444	A/Sachsen-Anhalt/1/2024

Table S1. Accession number (Gisaid) of influenza A(H1N1)pdm09 viruses isolated in Germany between 2009 and 2024

EPI_ISL_18881985	A/Berlin/46/2023	EPI_ISL_18831405	A/Berlin/58/2023
EPI_ISL_18881984	A/Baden-Wuerttemberg/47/2023	EPI_ISL_18869443	A/Baden-Wuerttemberg/21/2024
EPI_ISL_18881983	A/Brandenburg/11/2023	EPI_ISL_18869442	A/Baden-Wuerttemberg/1/2024
EPI_ISL_18881982	A/Rheinland-Pfalz/14/2023	EPI_ISL_18869441	A/Berlin/66/2023
EPI_ISL_18881981	A/Nordrhein-Westfalen/24/2023	EPI_ISL_18869440	A/Berlin/48/2023
EPI_ISL_18881980	A/Baden-Wuerttemberg/46/2023	EPI_ISL_18869439	A/Niedersachsen/39/2023
EPI_ISL_18881979	A/Berlin/67/2023	EPI_ISL_18839331	A/Mecklenburg-Vorpommern/16/2023
EPI_ISL_18869469	A/Niedersachsen/14/2024	EPI_ISL_18839330	A/Mecklenburg-Vorpommern/15/2023
EPI_ISL_18869468	A/Baden-Wuerttemberg/25/2024	EPI_ISL_18839329	A/Mecklenburg-Vorpommern/14/2023
EPI_ISL_18869467	A/Saarland/3/2024	EPI_ISL_18839328	A/Bayern/20/2023
EPI_ISL_18869466	A/Brandenburg/10/2024	EPI_ISL_18839327	A/Sachsen/5/2023
EPI_ISL_18869465	A/Brandenburg/9/2024	EPI_ISL_18839326	A/Sachsen/6/2023
EPI_ISL_18869464	A/Sachsen/3/2024	EPI_ISL_18839325	A/Hessen/44/2023
EPI_ISL_18869463	A/Nordrhein-Westfalen/17/2024	EPI_ISL_18839324	A/Hessen/43/2023
EPI_ISL_18869462	A/Hessen/12/2024	EPI_ISL_18839323	A/Baden-Wuerttemberg/45/2023
EPI_ISL_18869461	A/Baden-Wuerttemberg/24/2024	EPI_ISL_18839322	A/Baden-Wuerttemberg/44/2023
EPI_ISL_18869460	A/Baden-Wuerttemberg/23/2024	EPI_ISL_18839321	A/Saarland/5/2023
EPI_ISL_18869459	A/Thueringen/1/2024	EPI_ISL_18839320	A/Bayern/14/2023
EPI_ISL_18869458	A/Bremen/7/2024	EPI_ISL_18839319	A/Hessen/42/2023
EPI_ISL_18869457	A/Brandenburg/2/2024	EPI_ISL_18839318	A/Mecklenburg-Vorpommern/10/2023
EPI_ISL_18869456	A/Sachsen-Anhalt/2/2024	EPI_ISL_18839317	A/Mecklenburg-Vorpommern/13/2023
EPI_ISL_18869455	A/Bayern/11/2024	EPI_ISL_18839316	A/Mecklenburg-Vorpommern/12/2023
EPI_ISL_18869454	A/Hessen/11/2024	EPI_ISL_18839315	A/Mecklenburg-Vorpommern/9/2023
EPI_ISL_18869453	A/Bayern/10/2024	EPI_ISL_18839314	A/Bremen/4/2023
EPI_ISL_18869452	A/Nordrhein-Westfalen/16/2024	EPI_ISL_18839313	A/Thueringen/12/2023
EPI_ISL_18869451	A/Baden-Wuerttemberg/38/2023	EPI_ISL_18839312	A/Baden-Wuerttemberg/43/2023
EPI_ISL_18869450	A/Baden-Wuerttemberg/22/2024	EPI_ISL_18839311	A/Berlin/45/2023
EPI_ISL_18869449	A/Hessen/1/2024	EPI_ISL_18839310	A/Sachsen-Anhalt/14/2023
EPI_ISL_18839309	A/Sachsen-Anhalt/12/2023	EPI_ISL_18831408	A/Berlin/60/2023
EPI_ISL_18839308	A/Hessen/41/2023	EPI_ISL_18831407	A/Berlin/59/2023
EPI_ISL_18839307	A/Hessen/33/2023	EPI_ISL_18831406	A/Hessen/31/2023
EPI_ISL_18839306	A/Sachsen/4/2023	EPI_ISL_18831404	A/Berlin/57/2023

Table S1. Accession number (Gisaid) of influenza A(H1N1)pdm09 viruses isolated in Germany between 2009 and 2024

EPI_ISL_18839305	A/Baden-Wuerttemberg/42/2023	EPI_ISL_18576678	A/Mecklenburg-Vorpommern/4/2023
EPI_ISL_18839304	A/Baden-Wuerttemberg/33/2023	EPI_ISL_18831403	A/Berlin/56/2023
EPI_ISL_18839303	A/Rheinland-Pfalz/12/2023	EPI_ISL_18831402	A/Niedersachsen/24/2023
EPI_ISL_18839302	A/Baden-Wuerttemberg/41/2023	EPI_ISL_18831401	A/Berlin/42/2023
EPI_ISL_18839301	A/Brandenburg/9/2023	EPI_ISL_18831400	A/Brandenburg/8/2023
EPI_ISL_18839300	A/Rheinland-Pfalz/15/2023	EPI_ISL_18831399	A/Baden-Wuerttemberg/31/2023
EPI_ISL_18839299	A/Sachsen-Anhalt/13/2023	EPI_ISL_18831398	A/Bayern/19/2023
EPI_ISL_18839298	A/Berlin/65/2023	EPI_ISL_18831397	A/Rheinland-Pfalz/10/2023
EPI_ISL_18839297	A/Berlin/64/2023	EPI_ISL_18831396	A/Brandenburg/7/2023
EPI_ISL_18839296	A/Berlin/63/2023	EPI_ISL_18831395	A/Hessen/29/2023
EPI_ISL_18839295	A/Nordrhein-Westfalen/20/2023	EPI_ISL_18831394	A/Hessen/28/2023
EPI_ISL_18839294	A/Bremen/5/2023	EPI_ISL_18831393	A/Hessen/27/2023
EPI_ISL_18839293	A/Berlin/62/2023	EPI_ISL_18831392	A/Hessen/26/2023
EPI_ISL_18839292	A/Baden-Wuerttemberg/40/2023	*EPI_ISL_18831390*	*A/Saarland/3/2023*
EPI_ISL_18839291	A/Hessen/40/2023	EPI_ISL_19179545	A/Saarland/3/2023* (cell-cultured)
EPI_ISL_18832503	A/Berlin/50/2023	EPI_ISL_18831389	A/Berlin/41/2023
EPI_ISL_18831420	A/Berlin/51/2023	EPI_ISL_18831388	A/Niedersachsen/37/2023
EPI_ISL_18831419	A/Berlin/52/2023	EPI_ISL_18788624	A/Hessen/25/2023
EPI_ISL_18831418	A/Berlin/53/2023	EPI_ISL_18788621	A/Brandenburg/6/2023
EPI_ISL_18831417	A/Hessen/35/2023	EPI_ISL_18788619	A/Brandenburg/5/2023
EPI_ISL_18831416	A/Hessen/36/2023	EPI_ISL_18788617	A/Hessen/23/2023
EPI_ISL_18831415	A/Hessen/37/2023	EPI_ISL_18788616	A/Thueringen/9/2023
EPI_ISL_18831414	A/Hessen/38/2023	EPI_ISL_18788615	A/Niedersachsen/25/2023
EPI_ISL_18831413	A/Bremen/3/2023	EPI_ISL_18788614	A/Berlin/49/2023
EPI_ISL_18831412	A/Bremen/2/2023	EPI_ISL_18788613	A/Thueringen/8/2023
EPI_ISL_18831411	A/Bremen/6/2023	EPI_ISL_18788612	A/Baden-Wuerttemberg/39/2023
EPI_ISL_18831410	A/Nordrhein-Westfalen/15/2023	EPI_ISL_18788611	A/Rheinland-Pfalz/7/2023
EPI_ISL_18831409	A/Hessen/39/2023	EPI_ISL_18651955	A/Berlin/29/2023
EPI_ISL_18788609	A/Niedersachsen/23/2023	EPI_ISL_18651954	A/Berlin/30/2023
EPI_ISL_18651965	A/Berlin/39/2023	EPI_ISL_18651953	A/Brandenburg/4/2023
EPI_ISL_18651964	A/Berlin/34/2023	EPI_ISL_18576679	A/Nordrhein-Westfalen/11/2023
EPI_ISL_18651963	A/Berlin/36/2023	EPI_ISL_18576677	A/Schleswig-Holstein/5/2023

Table S1. Accession number (Gisaid) of influenza A(H1N1)pdm09 viruses isolated in Germany between 2009 and 2024

EPI_ISL_18651962	A/Berlin/35/2023	EPI_ISL_18651956	A/Berlin/31/2023
EPI_ISL_18651961	A/Mecklenburg-Vorpommern/5/2023	*EPI_ISL_18900980*	*A/Niedersachsen/39/2024*
EPI_ISL_18651960	A/Rheinland-Pfalz/8/2023	EPI_ISL_19179544	A/Niedersachsen/39/2024* (cell-cultured)
EPI_ISL_18651959	A/Berlin/33/2023	EPI_ISL_18514884	A/Berlin/26/2023
EPI_ISL_18651957	A/Berlin/38/2023	EPI_ISL_18788610	A/Saarland/4/2023

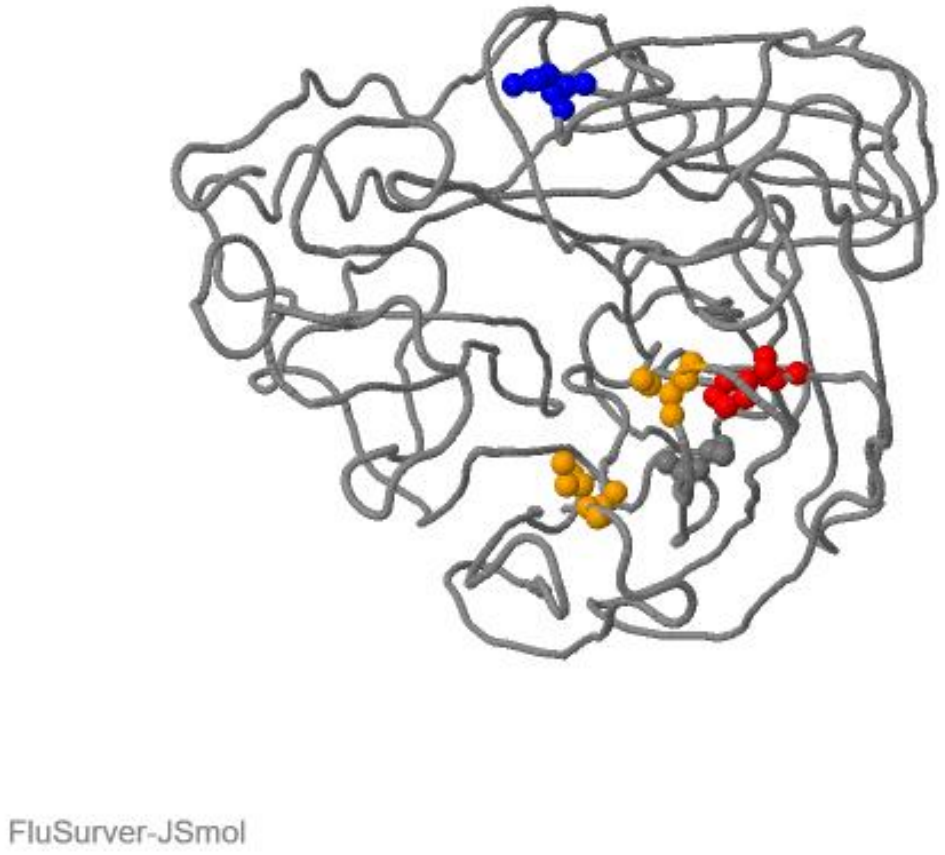

Figure S1: 3D-structure of A(H1N1)pdm09 neuraminidase A/California/07/2009-like with relevant amino acid substitutions included in yellow: I223V and S247N, black:V241I, blue: N369K, and red: H275Y.
The substitutions were manually inserted into the neuraminidase genome cDNA sequence of the reference virus A/California/7/2009 and the band structure was generated using BII FluSurver (https://flusurver.bii.a-star.edu.sg/).
